# Lymphatic filariasis in Papua New Guinea: distribution at district level and impact of mass drug administration, 1980 to 2011

**DOI:** 10.1186/1756-3305-6-7

**Published:** 2013-01-11

**Authors:** Patricia M Graves, Leo Makita, Melinda Susapu, Molly A Brady, Wayne Melrose, Corinne Capuano, Zaixing Zhang, Luo Dapeng, Masayo Ozaki, David Reeve, Kazuyo Ichimori, Walter M Kazadi, Frederick Michna, Moses J Bockarie, Louise A Kelly-Hope

**Affiliations:** 1 Department of Public Health, Tropical Medicine and Rehabilitation Sciences, James Cook University, Cairns and Townsville, Queensland, Australia; 2 Department of Health, Port Moresby, Papua New Guinea; 3 WHO, Port Moresby, Papua New Guinea; 4 Current address: WHO, Regional Office for the Western Pacific, Manila, Philippines; 5 WHO, Pacific Programme to Eliminate Lymphatic Filariasis (PacELF), Suva, Republic of Fiji; 6 WHO, Geneva, Switzerland; 7 Liverpool School of Tropical Medicine (LSTM), Centre for Neglected Tropical Diseases, Liverpool, United Kingdom of Great Britain and Northern Ireland; 8 James Cook University, PO Box 6811, Cairns, Queensland, 4870, Australia; 9 Current address: University of Alabama School of Medicine, Birmingham, Alabama, USA

**Keywords:** Lymphatic filariasis, Papua New Guinea, Mapping, Mass drug administration

## Abstract

**Background:**

Lymphatic filariasis (LF) caused by *Wuchereria bancrofti* is present at high prevalence in some parts of Papua New Guinea. However, there has been no rigorous data-based representative assessment of nationwide prevalence of LF. The LF programme has been daunted by the scope of the problem, and progress on mass drug administration (MDA) has been slow and lacking in resources.

**Methods:**

A systematic literature review identified LF surveys in Papua New Guinea between 1980 and 2011. Results were extracted by location, time period and test used (blood slide, immunochromatographic test (ICT) or Og4C3 ELISA) and combined by district. Three criteria schemes based on the Global Programme to Eliminate Lymphatic Filariasis guidelines, with modifications, were developed to classify and prioritize districts by prevalence level. Results of repeated surveys in the same sites were used to investigate the impact of MDA on LF prevalence over the time period.

**Results:**

There were 312 distinct survey sites identified in 80 of the 89 districts over the 31-year period. The overall LF prevalence in the sites tested was estimated at 18.5 to 27.5% by blood slide for microfilariae (Mf), 10.1% to 12.9% by ICT and 45.4% to 48.8% by Og4C3. Biases in site selection towards areas with LF, and change in type of assay used, affected the prevalence estimates, but overall decline in prevalence over the time period was observed. Depending on the criteria used, 34 to 36 districts (population 2.7 to 2.9 million) were classed as high endemic (≥5% prevalence), 15 to 25 districts (1.7 to 1.9 million) as low endemic (<5%) and 20 to 31 (1.3 to 2.2 million) as non-endemic. Nine districts (0.7 million) had no information. The strong impact of MDA, especially on microfilaria (Mf) prevalence, was noted in sites with repeat surveys.

**Conclusions:**

This analytical review of past surveys of LF in Papua New Guinea enables better estimation of the national burden, identifies gaps in knowledge, quantifies and locates the population at risk, and can be used to predict the likely impact of MDA and/or vector control. Better targeting of districts by level of prevalence will strengthen the control programme, facilitate monitoring of the disease trend and increase the likelihood of reaching the target of LF elimination by 2020.

## Background

Lymphatic filariasis (LF) is a mosquito-transmitted helminth infection, caused by *Wuchereria bancrofti* and transmitted predominantly by *Anopheles* mosquitoes in Melanesian countries in the Western Pacific. Papua New Guinea is often described as having very high prevalence of LF – perhaps the highest in the world [1,2]. Sites with very high prevalence have been found in Papua New Guinea, leading to much groundbreaking research on the dynamics of transmission, the relationship between infection and morbidity, and the effect of preventive chemotherapy, also known as mass drug administration (MDA) [3-7].

Lymphatic filariasis can be transmitted by a wide range of mosquito genera [8]. The main vectors of LF in Papua New Guinea are *Anopheles* mosquitoes (*An farauti, An punctulatus, An koliensis* and others) although *Culex* have also been found infected [9-12]. *Anopheles* also transmit malaria in the majority of the endemic LF districts of Papua New Guinea. Distribution of LF is known to be very heterogeneous, being affected by variation in altitude and possibly by former indoor residual spraying (IRS) campaigns for malaria, as was observed in the Solomon Islands and West Papua (formerly Netherlands New Guinea) [13,14]. Indoor residual spraying with dichlorodiphenyltrichloroethane) DDT occurred in the East Sepik and Madang Provinces of Papua New Guinea from the 1950s to the 1980s [15,16]. Mosquito nets (untreated with insecticide) have also been shown to be effective in reducing prevalence of LF [17-20]. Recently, malaria endemic areas have received large scale distributions of insecticide treated nets (ITN) in 2005–2006 and long lasting insecticidal nets (LLIN) in 2008–2011 [21].

### Prevalence of Lymphatic Filariasis in Papua New Guinea

There has been no rigorous data-based representative, comprehensive and recent assessment of nationwide prevalence of LF in Papua New Guinea. A summary of surveys for microfilariae in 46 sites in the New Guinea mainland and islands between 1912 and 1952 found prevalence varying from 0% to 71.4%, with a crude average of 26% [11]. Based on limited survey data from the 1970s and 1980s, Michael and Bundy [2] put Papua New Guinea in the 20 to 50% prevalence range using modeling and prediction. Kazura and Bockarie [1] summarized information available to 2003 by district and province, and stated that prevalence varied from 10% to 92% locally.

A preliminary national estimate of 6% LF prevalence in Papua New Guinea was obtained based on the LF Programme mapping prior to the first drug application to the Pacific Programme to Eliminate Lymphatic Filariasis (PacELF) [22]. Most of this initial mapping followed the WHO programme managers’ guidelines for preparing and implementing national plans to eliminate LF that were available at the time [23], which recommended lot quality assurance surveys (LQAS) of 250 older schoolchildren or adults in areas planned to be implementation units (IUs) where prevalence of LF was not known. However some other surveys and studies tested as few as 50–100 persons, as recommended in other WHO documents [24], in order to classify eligibility of areas for MDA. In both mapping protocols, a single positive (or more) for Mf or antigenemia was enough for an area to be classified as endemic for LF. At the onset of the LF Programme in Papua New Guinea, planned IUs were defined as provinces, although a significant proportion of mapping between 2000 and 2006 was conducted by district, with usually one site per district included.

### GPELF, PacELF and Papua New Guinea

The Global Programme to Eliminate Lymphatic Filariasis (GPELF) has the goal of interrupting LF transmission worldwide by 2020 using a strategy of preventive chemotherapy -- in this case, MDA for at least five years [25]. In 1999, the Pacific Programme to Eliminate Lymphatic Filariasis (PacELF), which included Papua New Guinea, was formed, with MDA starting in Samoa and Vanuatu shortly thereafter [22]. Most Pacific island countries and areas have made good progress towards the elimination goal, but the LF Programme in Papua New Guinea has suffered from limited resources, challenging terrain and logistical difficulties. Although Papua New Guinea has been the site of extensive research on LF control, this has often been done in localized areas and the results not assessed in a combined manner.

### Diagnostic tests for Lymphatic Filariasis

There are currently three recommended tests for *W. bancrofti* filariasis: examination of stained blood slides to detect Mf, the rapid immunochromatographic test (ICT), and the Og4C3 antigen ELISA assay using serum or plasma, including elution from dried blood spots. The tests were described and compared by Gass *et al*. [26]:

a) Blood slides: thick blood films are made using either i) 20 ul of fingerprick blood in a circle (as for a malaria thick blood film), ii) 60 ul of fingerprick blood in three lines on a slide using a haemocytometer pipette or a hematocrit tube, or iii) a larger amount of venous blood (1 to 10 ml) which is then passed through Millipore filters in the Knott’s or modified Knott’s methods [27]. Blood films on slides are not fixed before staining with Giemsa or similar and examining under x400 with identification of Mf as described in [28]. Filters, if used, are also stained and blood is examined on the filter under ×400. For detection of Mf in Papua New Guinea, blood must be taken at night to maximize the number of Mf present in peripheral blood. Although some Mf are present in the daytime in heavy infections, the infection is periodic and both Mf counts and estimated prevalence will be higher at night.

b) ICT card test: The rapid immunochromatographic test detects antigen from the adult worm that is circulating in the peripheral blood, using a card format and a coloured line readout. It detects higher prevalence in the population than Mf tests on blood slides, since a person may be infected with adult worms that are not producing Mf. The test uses 100 ul of blood. Originally developed in 1997 [29], the test is currently available from Binax NOW at Inverness Medical http://www.binaxnow.com/filariasis.aspx.

c) Og4C3 antigen ELISA: This is a laboratory-based test also detecting antigen from the adult worm. The test uses 50ul of serum or the equivalent eluted from dried whole blood spots. The test is available from TropBio http://www.tropbio.com.au.

### PNG LF programme

In 2011, Papua New Guinea had 18 provinces, plus the National Capital District and Autonomous Region of Bougainville. Two new provinces (Hela and Jiwaka) were formed in May 2012 by the splitting of Western Highlands, Enga and Southern Highlands provinces [30]. According to the National Research Institute report in 2010 [31], there were 89 districts and 231 local level government areas. The last census was in 2000, when the estimated population was 5.2 million [31]; the population in 2012 is estimated by one source to be 6.3 million based on an annual growth rate of 1.936% [32].

From baseline mapping surveys and using provinces as the IUs, the Papua New Guinea LF programme initially concluded that there were only four provinces or equivalent areas that were completely non-endemic (Eastern Highlands, Manus, Central and the National Capital District) and thus did not need MDA [33]. However, the decision to use the province as the IU became challenging, as the remaining population of 16 provinces needing to be covered in each round was huge, and the logistics of getting adequate coverage (at least 65% of total population) extremely difficult. At the Regional Programme Managers’ Meeting on Lymphatic Filariasis and other Selected Neglected Tropical Diseases convened by the WHO Regional Office for the Western Pacific in 2011 [34], it was recommended to change the IU from province to district in order to reduce the size of the target population, to prioritize the districts and to make the programme rollout more manageable with a community-based approach.

Research projects have shown that MDA has impacted LF prevalence dramatically in several provinces including Southern Highlands [3], Western [35,36], East Sepik [4,6,7], Madang [37-39] and New Ireland [40]. These studies have often been done in limited areas for a few years only, and in some sites five rounds of MDA did not reduce prevalence to below criteria for stopping MDA [7,36,40,41]. However, they provide important information on the impact of MDA and the combined evidence from these studies needs to be comprehensively assessed.

In addition to the research studies mentioned above, one-time province-wide MDAs have also been conducted in several provinces in conjunction with LLIN distribution. This was done in 2005 and 2006 in the Autonomous Region of Bougainville, Oro (Northern), Gulf, New Ireland, East and West New Britain, East Sepik and Morobe provinces, but was found not to be a sustainable mechanism for MDA because LLINs are not distributed annually. Other MDAs were carried out in Milne Bay Province, which conducted three rounds of MDA in 2005–2007 (including one with LLIN distribution) and two more rounds in 2010 and 2011. Despite these achievements, generally the challenges of delivering MDA and monitoring transmission in Papua New Guinea have been enormous, resources have been extremely limited and other priorities including malaria and HIV have predominated.

A new approach is needed to get Papua New Guinea back on track for elimination by 2020 in line with the global target [42]. Given the heterogeneity of transmission, it is likely that large areas without any LF are being targeted for MDA, while logistical and political issues mean that some areas with higher prevalence are being neglected. Therefore, revitalization of the LF Programme and initiation of MDA nationwide in time to meet the 2020 target is urgently needed, and a thorough and up- to-date review of LF prevalence level by district may assist in the prioritization of human and financial resources to overcome these barriers.

In this study, data were gathered for all locations where surveys have been done (separately for ICT, Mf and Og4C3 tests). Using this information, areas still to be mapped were identified, districts with high prevalence were noted for prioritization and the size of the population at risk was re-evaluated. District endemicity was classified by three different criteria and new empirical risk maps for Papua New Guinea were developed. Changes in prevalence over time (using three time periods) and the potential impact and benefits of MDA were also assessed.

## Methods

A search of PubMed was done using the terms (‘Papua New Guinea’ or ‘New Guinea’) and (‘Lymphatic Filariasis’ or ‘*Wuchereria bancrofti*’ or ‘*W.bancrofti*’ or ‘filariasis’ or ‘elephantiasis’). Reference lists of published studies were searched for additional references. Informal James Cook University survey reports and relevant doctoral theses at James Cook University, the University of Papua New Guinea and the University of Queensland were retrieved and searched for survey results and locations. Papua New Guinea Department of Health reports, PacELF and WHO meeting reports and records, and MDA drug applications from 1999 (the start of PacELF) to date were also examined.

Data on surveys in Papua New Guinea since 1980 were extracted with locations, numbers tested, numbers positive, test used, age group, and method of Mf examination, where available. Research studies testing interventions (mostly MDA, but some mosquito net projects) were extracted separately by village and time period where possible. Occurrence of any MDA or number of MDA rounds in locations of all surveys was noted, if given or available from other sources. Coordinates of unknown locations were obtained from the Geographic Names Server http://earth-info.nga.mil/gns/html and/or Global Gazetteer Version 2.2 http://www.fallingrain.com/world. Locations were assigned to districts using the 2010 district and provincial profiles from the National Research Institute of Papua New Guinea [31]. Mapping of prevalence estimates by district was done using the geographical information system (GIS) shape files developed by the University of Papua New Guinea Remote Sensing Centre, available at http://gis.mortxonblacketer.com.au/upngis/instructions.htm.

Districts were classified as non-endemic, endemic or unknown by three different criteria schemes and the results compared. The first criteria scheme was based on the Global Programme to Eliminate Lymphatic Filariasis (GPELF) and the second two schemes based on modified or alternative criteria, which also classified districts into low endemic and high endemic. The criteria schemes were as follows:

1. GPELF criteria:

Considering only the most recent mapping survey (target 250 persons tested) by any test in each district:

Zero positives = non endemic

Any positives = endemic

2. GPELF modified criteria:

Considering any test in each district:

Zero positives = non-endemic

If >0% to <5% positive = low endemic

If ≥5% positive = high endemic

Where an unknown district is bordered on all sides by endemic districts, classify as ‘low endemic’ or ‘high endemic’ based on lowest category found in adjacent districts.

3. Alternative criteria:

Using data from all surveys, using Mf results if available, then ICT or Og4C3 results if no Mf tests performed in that district. Considering all positives for the selected test:

If <1% positive = non-endemic

If ≥1% to <5% positive = low endemic

If ≥5% positive = high endemic

## Results

The literature search identified 324 separate survey sites between 1980 to 2011 that are listed by site and date in Additional file 1: Table S1. Results of 12 survey sites at the same location and time that were reported in duplicate (usually summarized in different ways), were identified and removed (and noted in Additional file 1: Table S1), leaving 312 separate survey site data points for analysis. The source of information, location, date (year), type of assay, the amount of blood tested (for Mf), the time of testing (for Mf) and the age group tested were noted for each survey, where available. It was also noted whether any MDA, and if possible how many MDAs and which drug used, had been conducted in the locality prior to the survey.

A survey site represents a distinct named geographic location tested at a point in time. This may have been a small number of households in a hamlet that formed part of a larger group in a village survey. The number of persons tested per site ranged from 6 to 1666 for Mf (mean 211 per site), from 1 to 3799 for ICT (mean 290) and from 9 to 1322 for Og4C3 (mean 209). There were 155 surveys that used Mf, 149 using ICT and 79 using Og4C3. The total comes to more than the overall number of survey sites, since some surveys used more than one test. There was no district that had only Og4C3 surveys; therefore districts were classified by endemicity using Mf and ICT results only.

### Estimates of national prevalence of Lymphatic Filariasis

Combining all the surveys for each assay type gave the following estimates:

Mf: 27.5%, ICT: 12.9%, Og4C3: 48.8% (Table 1). Since the results are biased by the differing sample sizes and the fact that more surveys were done in research sites with high prevalence of Mf, an alternative estimate was made by crudely averaging the prevalence in each district (Table 1). These estimates were:

**Table 1:** Summary percent positive for LF (microfilariae or antigenemia) by district (all surveys conducted 1980–2011)

**REGION**	**DISTRICT**	Pop 2010 est*	**No of sites surveyed Mf**	**% pos Mf**	**N tested Mf**	**No of sites surveyed ICT**	**% pos ICT**	**N tested ICT**	**No of sites surveyed Og4C3**	**% pos Og4C3**	**N tested Og4C3**	**Endemic GPELF Criteria**	**Endemic mod GPELF Criteria**	**Endemic Alt Criteria**
Bougainville autonomous region	Central Bougainville	48145				3	4.3	694	1	0	415	Yes	Low	Low
	North Bougainville	84825	3	20.2	322	1	12.8	218	3	64.7	331	Yes	High	High
	South Bougainville	70310				1	5.0	280				Yes	High	High
Central	Abau	48177				1	0	250				No	No	No
	Goilala	34327				1	0	250				No	No	No
	Kairuku-Hiri	98900				4	0	1062				No	No	No
	Rigo	49555				4	0	768				No	No	No
Chimbu (Simbu)	Chuave	43545				1	0	250				No	No	No
	Gumine	43426				1	0.8	250				Yes	Low	No
	Karimui-Nomane	43859										?	?	?
	Kerowagi	66209				1	2.4	250				Yes	Low	Low
	Kundiawa-Gembogl	70560				1	0	250				No	No	No
	Sina Sina-Yonggomugl	45888				1	0	250				No	No	No
East New Britain	Gazelle	114921				2	12.8	500				Yes	High	High
	Kokopo	74687	2	22.6	115	3	28.8	697	2	62.8	419	Yes	High	High
	Pomio	57558	3	11.1	262	3	25.3	403	3	45.1	366	Yes	High	High
	Rabaul	34624					24.4	250				Yes	High	High
East Sepik	Ambunti-Dreikikir	68864	48	33.8	19235				13	68.0	3488	Yes	High	High
	Angoram	85521	1	1.1	90							Yes	Low	Low
	Maprik	71859	1	1.0	200							Yes	Low	Low
	Wewak	79515				3	25.7	1086				Yes	High	High
	Wosera Gawi	61419				1	1.0	200				Yes	Low	Low
	Yangoro Saussia	59432				1	4.1	121				Yes	Low	Low
Eastern Highlands	Daulo	38487				1	0	250				No	No	No
	Goroka	89342				1	0	250				No	No	No
	Henganofi	69326				1	0	250				No	No	No
	Kainantu	114081				1	0	250				No	No	No
	Lufa	57019				1	0	250				No	No	No
	Obura-Wonenara	36905				2	0	750				No	No	No
	Okapa	77124				1	0	250				No	No	No
	Unggai-Benna	55947				2	0	750				No	No	No
Enga	Kandep	63086										?	?	?
	Kompiam	59003				1	0	186				No	No	No
	Lagaip-Porgera	121117				1	0	113				No	No	No
	Wabag	78192				1	0.98	205				Yes	Low	No
	Wapen-amanda	71267										?	?	?
Gulf	Kerema	84665	2	19.3	688	2	30.2	716	1	64.9	222	Yes	High	High
	Kikori	53515				1	2.8	250				Yes	Low	Low
Madang	Bogia	74537				1	1.0	100				Yes	Low	Low
	Madang	113159	1	32.3	96							Yes	High	High
	Middle Ramu	75548	1	41.5	106	1	13.0	92	3	35.5	262	Yes	High	High
	Rai Coast	73486				1	10.3	68				Yes	High	High
	Sumkar	87522	4	23.9	2032				3	49.5	1690	Yes	High	High
	Usino-Bundi	52314	4	7.8	2510	4	31.2	2488				Yes	High	High
Manus	Manus	56083				1	0	250				No	No	No
Milne Bay	Alotau	95551	8	21.1	479	6	32.6	988	5	57.5	1101	Yes	High	High
	Esa’ala	54588				3	55.4	1250				Yes	High	High
	Kiriwina-Goodenough	63961				3	20.0	1076				Yes	High	High
	Samarai-Murua	55246	4	40.0	400	6	7.4	2187	7	24.0	1199	Yes	High	High
Morobe	Bulolo	101795				1	30.2	149				Yes	High	High
	Finschafen	59690				1	30.0	250				Yes	High	High
	Huon	78454				1	29.7	128				Yes	High	High
	Kabwum	55204				1	30.4	115				Yes	High	High
	Lae	157082				1	30.1	226				Yes	High	High
	Markham	65071				1	13.2	250				Yes	High	High
	Menyamya	90347				1	6.0	100				Yes	High	High
	Nawae	46183				1	28.0	250				Yes	High	High
	Tewae-Siassi	57107	1	2.7	150							Yes	Low	Low
National Capital District	Moresby North East	120664**				2	6.2	341				Yes	High	High
	Moresby North West	120664										?	?	?
	Moresby South	120666				6	1.4	1019				Yes	Low	Low
New Ireland	Kavieng	71099	4	15.5	380	2	12.5	767	4	55.5	418	Yes	High	High
	Namanatai	86416	16	20.0	2029	6	9.8	11884	6	44.9	1307	Yes	High	High
Northern (Oro)	Ijivitari	88727	1	0	402	2	2.0	702	1	3.1	416	Yes	Low	No
	Sohe	84961	1	0	462	2	0.9	750	1	1.0	484	Yes	Low	No
Southern Highlands	Ialibu-Pangia	76654				1	0	250				No	No	No
	Imbonggu	90667				1	1.9	213				Yes	Low	Low
	Kagua-Erave	82261										?	?	?
	Komo-Margarima	96818										?	?	?
	Koroba-Kopiago	104986										?	?	?
	Mendi-Munihu	145483				1	0.8	250				Yes	Low	No
	Nipa-Kutubu	148640	7	39.8	771				1	51.9	181	Yes	High	High
	Tari-Pori	78783										?	?	?
West New Britain	Kandrian-Gloucester	79356	3	2.5	355	4	19.5	523	4	55.4	417	Yes	High	Low
	Talasea	183437	2	2.9	377	1	6.1	412	2	22.9	455	Yes	High	Low
West Sepik (Sandaun)	Aitape-Lumi	67670										?	Low	?
	Nuku	58339				1	3.7	136				Yes	Low	Low
	Telefomin	45112				1	10.9	193				Yes	High	High
	Vanimo-Green River	64335				1	22.0	250				Yes	High	High
Western (Fly)	Middle Fly	77277	3	41.4	1138	2	69.4	674	15	53.0	2374	Yes	High	High
	North Fly	70443	35	25.8	4826				1	57.0	676	Yes	High	High
	South Fly	64388				2	0.4	500				Yes	Low	No
Western Highlands	Anglimp-South Wahgi	123618				1	0.4	250				Yes	Low	No
	Dei	63706				1	2.4	250				Yes	Low	Low
	Jimi	47856				1	0	234				No	No	No
	Mount Hagen	111305				2	0.9	450				Yes	Low	No
	Mul-Baiyer	72563				1	0.8	250				Yes	Low	No
	North Wahgi	66363				1	0.8	250				Yes	Low	No
	Tambul-Nebilyer	77859				1	0.8	250				Yes	Low	No
Grand Total		6829240	167	27.5	37425	148	12.9	43264	79	48.8	16221			
Crude average of sites				18.5			10.1			45.4				

* obtained from source [31] with adjustment of 2000 census district figures by province- specific growth rates 1980–2000.

** population of National Capital District equally divided between three districts.

Mf: 18.5%, ICT: 10.1%, and Og4C3: 45.4%. While these lower estimates do not eliminate the bias due to surveys being done in sites where LF was expected to be found, they represent the best estimates in the absence of a national survey with representative sampling.

### Comparison between diagnostic tests

When used on the same population and time point, Mf tests are expected to give lower percent prevalence estimates than serological tests that detect adult worm antigen (ICT or Og4C3) [26]. However, in Table 1 it can be seen that lower prevalence by Mf is not always observed for the summarized district data or overall (see previous paragraph), as a result of combining surveys done in different locations and times. Detailed comparison of diagnostic tests is beyond the scope of this paper and will be reported separately, but it can be seen in Additional file 1: Table S1 that the expected relationships between tests are observed when considering only those sites that used more than one test at the same survey. Prevalence estimated by Mf was always lower than ICT prevalence (19 comparisons) and Mf prevalence only exceeded Og4C3 prevalence for one survey in 1994 (total of 47 comparisons).

### Surveys classified by time period

**Figure 1:**
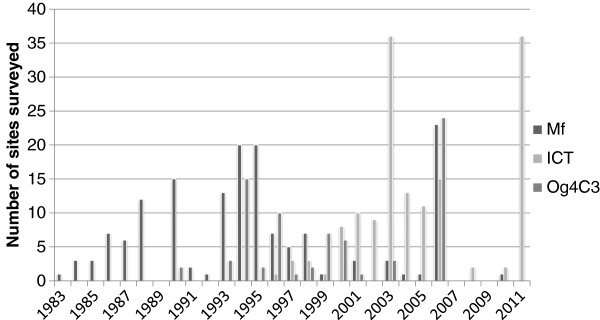
Number of individual survey sites by year, classified by assay type.

**Figure 2:**
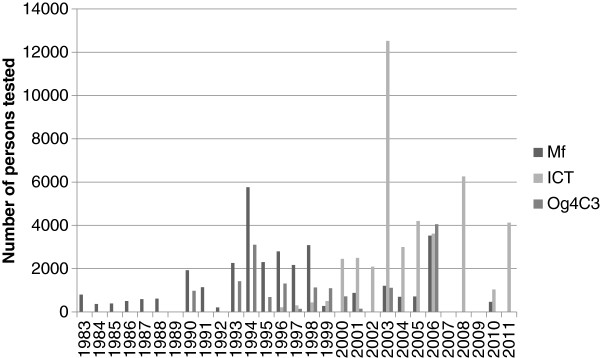
Number of persons tested by year, classified by assay type.

The number of sites surveyed by year is shown in Figure 1 while the number of persons tested per year is shown in Figure 2. The first survey identified was in 1983. Three years (1989, 2007 and 2009) had no surveys by any test. All surveys prior to 1990 detected Mf in blood, while the majority after 2000 used ICT. Og4C3 tests started in 1990 while the first ICT tests were done in 1996.

Surveys were divided into approximately three equal time periods: 1983–1992 (10 years), 1993–2002 (10 years) and 2003–2011 (9 years), to investigate changes over time. There was a decline in the percentage positive in Mf and Og4C3 tests over the three periods (Table 2), but no obvious decline in ICT percentage positive in the latter two time periods.

**Table 2:** Summary of survey results by three time periods

	**Mf**			**ICT**			**Og4C3**		
	**No sites**	**No persons**	**% pos**	**No sites**	**No persons**	**% pos**	**No sites**	**No persons**	**% pos**
1983–1992	50	6539	30.4	0	0		2	976	64.7
1993–2002	76	19540	30.1	35	8502	13.4	47	9755	56.9
2003–2011	29	6608	7.8	115	34762	12.8	27	5169	28.9

### Overall classification of districts and mapping

**Figure 3:**
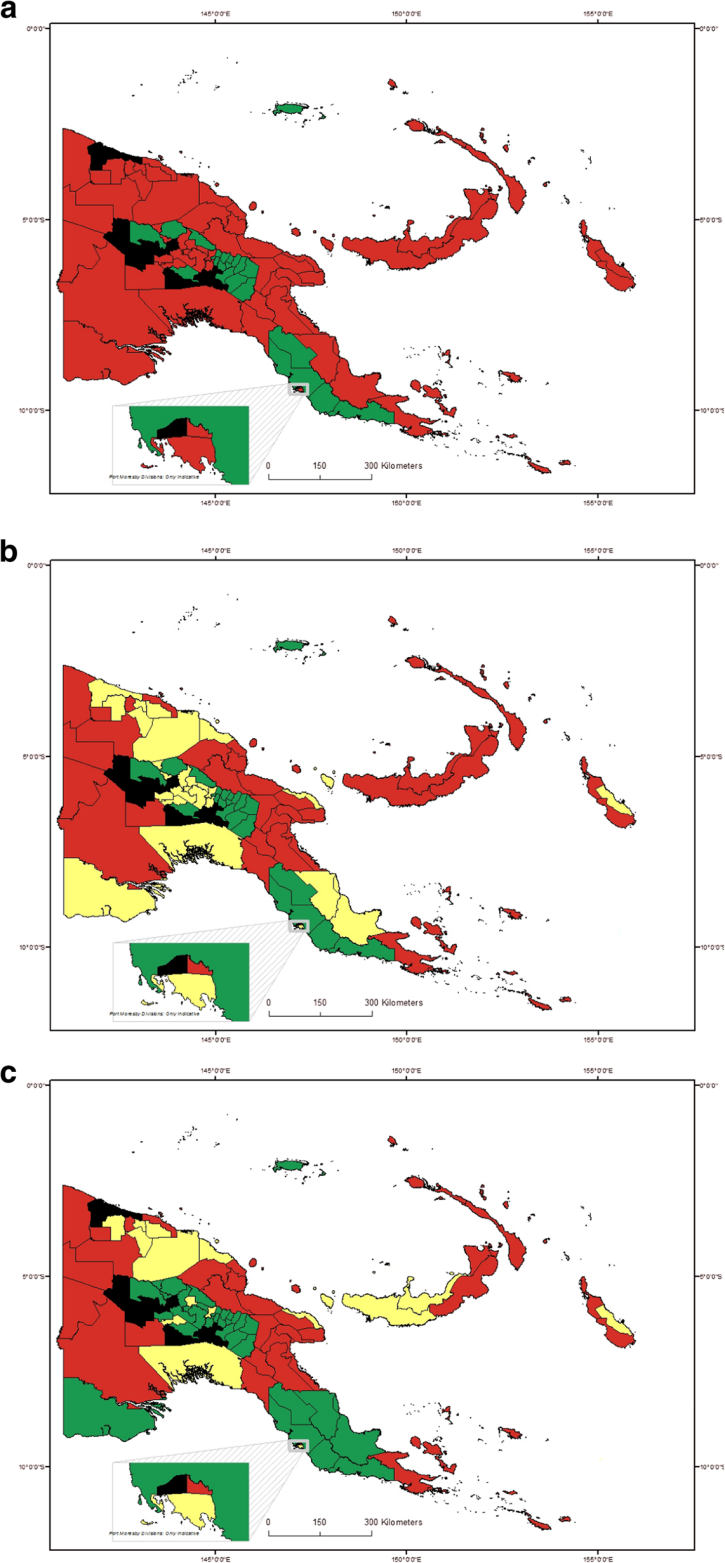
**Maps showing classification of districts by endemicity, according to three criteria schemes. a**. Map with districts classified using GPELF criteria scheme 1. Red: endemic, >0% pos; Green: non-endemic, 0% pos; Black: unknown; results from all types of test. **b**. Map with districts classified using modified GPELF criteria scheme 2. Red: High endemic, ≥5% pos; Yellow: Low endemic, >0% and <5% pos (or unknown but all adjacent districts >0%); Green: non-endemic, 0% pos; Black: unknown; results from all types of test. **c**. Map with districts classified using alternative criteria scheme 3. Red: high endemic; ≥5% pos; Yellow: low endemic, ≥1% and <5% pos; Green: non-endemic, <1% pos; Black: unknown; Mf results used if available, otherwise ICT.

Data were summarized by district for all surveys in each district, by test, and are shown in Table 1. Using the three sets of criteria described in the methods section, numbers of districts in each category are shown in Table 3. Figures 3a, 3b and 3c show the classification of endemicity by district, shaded according to the three criteria schemes described above.

**Table 3:** Classification of districts by three criteria schemes

	**1 GPELF criteria**	**2 GPELF modified criteria**	**3 Alternative criteria**
High endemic	60	36	34
Low endemic		25	15
Non endemic	20	20	31
Unknown	9	8	9
Total districts	89	89	89

Overall the picture is similar for most districts by any criteria scheme, in that lowland and coastal districts were more likely to be endemic than inland highland districts. A major distinction between the schemes is that criteria scheme 1 gives only a ‘yes or no’ classification of prevalence, whereas both criteria schemes 2 and 3 divide prevalence into 3 levels – non-endemic, low and high. Criteria schemes 1 (GPELF) and 2 (GPELF modified) classify many more districts as endemic compared to the criteria scheme 3 (alternative), mainly because in the first two schemes, a single positive by any test classifies a district as endemic (regardless of sample size). In the alternative criteria scheme 3, ≥1% of tested persons must be positive (in Mf surveys if available; if not then ICT, with all surveys over time combined) in order for a district to be classified as endemic.

No surveys have been conducted to date in nine districts (of 89 total), in the following provinces: Chimbu (one district), Enga (two districts), NCD (one district), Southern Highlands (four districts), and West Sepik (one district). The unknown district in West Sepik (Aitape Lumi) was surrounded by endemic districts, and therefore classified under criteria scheme 2 as low endemic based on level of endemicity in neighbouring districts. The other unknown districts were each adjacent to both non-endemic and endemic districts and therefore have not been classified.

### Impact of MDA on prevalence of Lymphatic Filariasis

It was known whether or not MDA had occurred prior to the survey in all except one case (see Additional file 1: Table S1). The majority of the surveys occurred prior to any MDA. The number of MDAs at each site was also obtained from the available studies and reports.

**Figure 4:**
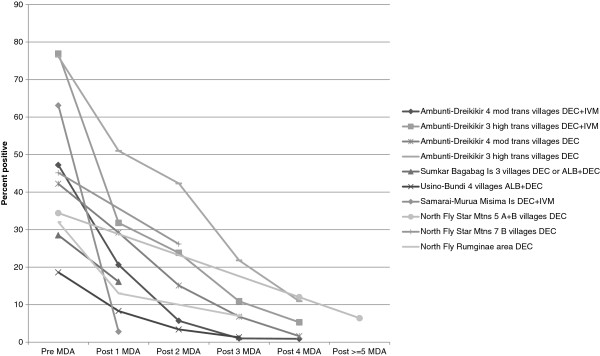
Percentage of persons positive for Mf in sites surveyed more than once, according to number of rounds of MDA in that location.

**Figure 5:**
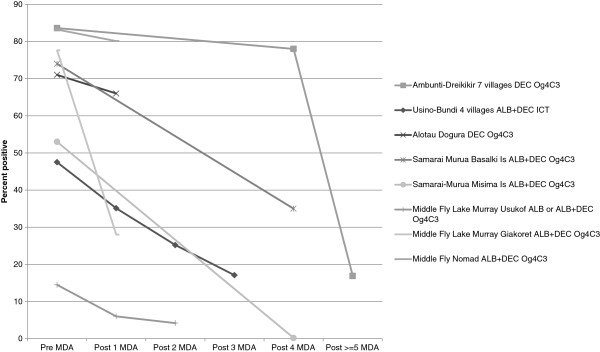
Percentage of persons positive for LF antigenaemia in sites surveyed more than once, according to number of rounds of MDA in that location.

In order to examine whether the decline in percentage positivity over time, which is apparent in Table 2 as noted above, was due to MDA or other interventions, sites with repeated surveys were categorized into groups having had 0, 1, 2, 3, 4 or > =5 MDAs prior to the survey (Table 4). The location, serological test used and MDA drug type and frequency are shown with the LF prevalence results from these sites in Table 4 for the appropriate time periods. The impact on prevalence of annual or twice a year MDA is shown graphically for Mf assays in Figure 4 and for ICT and Og4C3 assays in Figure 5. The impact on Mf prevalence was found to be very rapid and large (Figure 4) whereas the decline in antigen prevalence appeared to be slower (Figure 5).

**Table 4:** Summary of pre and post MDA survey results

**Province**	**District**	**Locality**	**Test**	**MDA drug**	**Frequency (if > =1 MDA)**	**Pre-MDA**			**Post 1 MDA**			**Post 2 MDA**			**Post 3 MDA**			**Post 4 MDA**			**Post > =5 MDA**			**Reference**
						**Yr**	**No pers**	**% pos**	**Yr**	**No pers**	**% pos**	**Yr**	**No pers**	**% pos**	**Yr**	**No pers**	**% pos**	**Yr**	**No pers**	**% pos**	**Yr**	**No pers**	**% pos**	
E Sepik	Ambunti-Dreikikir	4 mod trans villages	Mf	DEC + IVM	Annual	1994	797	47.2	1995	756	20.6	1996	790	5.7	1997	819	1.0	1998	750	0.9				[4]
E Sepik	Ambunti-Dreikikir	3 high trans villages	Mf	DEC + IVM	Annual	1994	281	76.9	1995	318	31.8	1996	311	23.8	1997	303	10.9	1998	266	5.3				[4]
E Sepik	Ambunti-Dreikikir	4 mod trans villages	Mf	DEC	Annual	1994	903	42.2	1995	815	29.2	1996	802	15.1	1997	692	6.8	1998	639	1.6				[4]
E Sepik	Ambunti-Dreikikir	3 high trans villages	Mf	DEC	Annual	1994	243	76.1	1995	192	51.0	1996	253	42.3	1997	257	21.8	1998	165	10.9				[4]
E Sepik	Ambunti-Dreikikir	7 villages	Og4C3	DEC	Annual	1994	177	83.6										1998	100	78	2003	531	16.9	[7]
Madang	Sumkar	Bagabag Is	Mf	DEC or ALB + DEC		1998	1026	28.5	2001	729	16.1													[43][37]
Madang	Usino-Bundi	4 villages	Mf	ALB + DEC	Annual	2003	571	18.6	2004	696	8.3	2005	714	3.4	2006	529	1.3							[39]
Madang	Usino-Bundi	4 villages	ICT	ALB + DEC	Annual	2003	558	47.5	2004	692	35.1	2005	695	25.2	2006	543	17.1							[39]
Milne Bay	Alotau	Buhutu valley	Og4C3	DEC	Salt (for 6 months)	1995	434	55	1996	100	36.3													[44,45]
Milne Bay	Alotau	Dogura	Og4C3	DEC		1995	255	71	1996	100	66													[44,45]
Milne Bay	Samarai-Murua	Basalaki Is	Og4C3	ALB + DEC	Annual	1998	100	74										2003	100	35				[46]
Milne Bay	Samarai-Murua	Misima Is	Mf	DEC + IVM	Annual	1996	100	63.1	1997	100	2.8													[46]
Milne Bay	Samarai-Murua	Misima Is	Og4C3	ALB + DEC	Annual	1997	144	53										2003	484	0.2				[46]
New Ireland	Namanatai	Lihir Is / E Coast	ICT	ALB + DEC								2003	3009	7.7				2008	3799	0.8				[40]
New Ireland	Namanatai	Lihir Is / W Coast	ICT	ALB + DEC								2003	1969	30.7				2008	2464	7.5				[40]
S Highlands	Nipa-Kutubu	Mt Bosavi: Fogomaiyu	Mf	DEC	Weekly (for 6 mths) + ITN	1987	79	92													1987	100	6	[3]
Western	Middle Fly	Lake Murray: Usukof	Og4C3	ALB or ALB + DEC	Bi-annual	1999	62	14.5	1999	168	6	2000	168	4.2										[47]
Western	Middle Fly	Lake Murray: Giakoret	Og4C3	ALB + DEC		1999	67	77.6	2000	25	28													[47]
Western	Middle Fly	Nomad	Og4C3	ALB + DEC		1999	262	83.2	2000	146	80.1													[47]
Western	North Fly	Star Mtns A + B villages	Mf	DEC	Bi-annual (for 2 yrs) then annual (for 2 yrs)	1986	286	34.4										1988	302	12	1990	503	6.4	[48]
Western	North Fly	Star Mtns B villages	Mf	DEC	Annual	1988	312	45.14				1990	372	26.14										[48]
Western	North Fly	Rumginae area	Mf	DEC	Annual	1991	1034	32	1992	208	13				1993	248	7							[49]

Mf: microfilariae; DEC: diethylcarbamazine; IVM: ivermectin; ALB: albendazole.

Three studies in Table 4 are not plotted in Figures 4 or 5 since they did not have an annual or biannual MDA schedule or consistent test used for evaluation:

1) The Milne Bay/Alotau district study by Sapak *et al*. [44], which used diethylcarbamazine (DEC) salt for six months and tested by Og4C3 antigen;

2) The Milne Bay/Samarai-Murua district/Lihir Island studies, where baseline surveys before MDA were done using Mf tests by Hii *et al*. [50] whereas follow-ups after 2 and 4 MDAs were done with ICT tests by MItja *et al*. [40].

3) The Southern Highlands/Nipa-Kutubu district study of Prybylski *et al*. [3], which used a schedule of weekly DEC for 6 months, and tested by Mf surveys.

## Discussion

Survey results showed large variation in prevalence over the country, which is in line with current research findings and historical data dating back to 1912 [11]. Such heterogeneity across the country is presumably related to the diverse geography, altitude and ecology in this unique country. The differences may also be explained by the diversity of mosquito vectors found in different regions and their ability to transmit. *Anopheles* vectors have been shown to be more efficient in transmission than *Culex* in Papua New Guinea [9], but there are major gaps in the current knowledge of LF vectors and intensity of transmission (except in one or two small areas) as well as on the impact of vector-based interventions. Variation in prevalence may be related to previous malaria control activities, including past insecticide spraying and more recently the distribution of mosquito nets of all types, as well as to some extent, MDA for LF in a limited number of sites.

The data presented and summarized in this study are potentially not representative of the true situation in Papua New Guinea because some survey sites were purposively selected to identify LF where it was thought to exist, and the results from the different surveys are not weighted. Therefore the prevalence estimates should be regarded as likely to be overestimates for the country as a whole. Despite these limitations, the survey results show generally lower prevalence of LF than is usually stated for Papua New Guinea, which is encouraging for the LF programme’s potential success. In addition, despite potential biases in site selection and assays used over time, the review suggests a clear decreasing trend in prevalence over the three broad time periods (Table 2).

The highest prevalence estimates were consistently found in the coastal provinces, especially those in the north and west of the country, before the year 2000. This is as expected given the warmer temperatures and higher humidity in these low-lying areas favoring mosquito transmission. Some regions, including the highland provinces and more developed areas near Port Moresby or around mine sites, tend to have lower prevalence particularly since 2000, and may be a reflection of the influence of urbanization (which is more unfavourable to *Anopheles* vectors) and climate as well as control measures.

The spatial heterogeneity of LF observed here in Papua New Guinea is typical of the disease worldwide. There is no doubt that similar records of past surveys exist in other countries and regions, and the compilation and mapping of such data in a systematic way including details on tests used, persons sampled and up-to-date population estimates would enable better prioritization of MDA efforts. Such assessments would assist in monitoring progress and efficacy of the global LF elimination programme and accelerate its achievements.

Many of the surveys included in this analytical review were conducted as part of research, or before GPELF mapping criteria were formulated. Therefore, it is difficult to apply the standard GPELF criteria or threshold for endemicity (one or more positives per 250 persons sampled) to such data. The GPELF modified criteria scheme 2 included in this study aimed to stratify the level of risk more finely than just non-endemic/endemic. The third alternate criteria scheme was developed to classify a district as negative if prevalence was less than 1% (rather than 0%), since many districts had sampled more than the minimum number needed for Lot Quality Assurance Sampling (LQAS) mapping, and to give more weight to Mf rather than antigen surveys. This review has identified nine districts that remain to be classified, although one (in West Sepik) is likely endemic since it is surrounded on all borders by endemic districts.

Previous estimates stated that five million people in Papua New Guinea are at risk of LF [33] out of a total population estimated to be between 6.3 and 6.8 million [31,32]. Using the LF district classifications gathered here, together with population estimates for 2010 by district (generated from the 2000 census using the province-specific growth rates from 1980–2000 [31]), we can quantify the number of persons potentially at risk of LF in Papua New Guinea who need MDA or other intervention to interrupt LF transmission, as follows:

1. By GPELF criteria,

• 4.81 million people (70.4% of the population) live in 60 ‘endemic’ districts;

• 0.73 million (10.7%) live in the nine unknown districts.

2. By modified GPELF criteria,

• 2.94 million (43.1% of the population) live in 36 ‘high endemic’ districts;

• 1.94 million (28.3%) live in 25 ‘low endemic’ districts;

• 0.66 million (9.7%) live in the eight unknown districts.

3. By alternative criteria,

• 2.68 million (39.2% of the population) live in 34 ‘high endemic’ districts;

• 1.17 million (17.2%) live in 15 ‘low endemic’ districts;

• 0.73 million (10.7%) live in the nine unknown districts.

Based on these different criteria schemes, we can rule out from MDA at least 20 districts (1.29 million people), and possibly as many as 40 districts (2.98 million people). Twenty is the number of non-endemic districts as assessed by the least specific GPELF Criteria Schemes 1 and 2, while under the alternative criteria scheme 3, a total of 31 districts are classified as non-endemic plus potentially nine more (those with currently unknown prevalence). Under criteria scheme 3, the priority ‘high endemic’ districts for MDA have a population of ‘only’ 2.68 million, compared to 4.81 million people in the endemic districts under criteria scheme 1.

The classification of districts will guide the LF programme towards the highest prevalence areas and leave the lower or uncertain areas until last, to maximize impact and avoid wasting resources in a country with significant geographical challenges and limited transport infrastructure. The high endemic districts (numbering 36 and 34 under criteria schemes 2 and 3 respectively) should be the most important focus, followed by the low endemic districts (25 and 15 respectively). Prioritizing high endemic districts will increase the LF programme’s ability to deliver MDA, which as this collation of data clearly highlights has a major impact on prevalence and transmission.

It should be noted that the information available for some districts is limited (one survey site), and it is possible that some may have been wrongly classified as non-endemic. Further surveys in such districts may be warranted when reports of LF morbidity, especially in young people, are received from health workers. It is also important to consider that many districts are large in geographical area (e.g. in Western Province), and thus potentially have areas within them that have different transmission intensity. All districts comprise a number of smaller administrative units called local level governments (LLGs). To date, some survey sites have only been mapped to district, but once survey points are all individually geo-located, it will be possible to classify endemicity of LLGs in the same way as districts and identify further subdistrict areas not needing MDA. The survey point locations data can also be used to model risk factors for infection (e.g. altitude, malaria risk, net coverage, population density, proximity to water) in future.

The MDA impact studies were undertaken in areas with different levels of endemicity and different ecological settings, which further supports the likelihood of successful elimination in Papua New Guinea, especially if coupled with vector control such as the recent scale up of LLIN distribution across many districts. The fact that LF is transmitted mainly by *Anopheles* vectors may be an advantage as this genus is more likely than *Culex, Mansonia* or *Aedes* to be impacted with traditional insecticide-based control methods such as ITNs, LLINS and IRS in an integrated vector management strategy as is currently being promoted by WHO [51]. A recent review has also advocated integrated vector management for malaria and LF control and highlighted the potential synergistic impact on both diseases [19].

Above all, this review of data to date highlights the gaps in data and our knowledge, such as the need to classify the remaining nine unknown districts. The greatest need is to mobilize a critical mass of in-country support and resources from interested funding agencies and international stakeholders with the aim to eliminate LF in Papua New Guinea by 2020.

## Conclusions

This analytical review of past surveys of LF in Papua New Guinea has enabled the country to more accurately estimate the national burden of LF, identify gaps in knowledge and predict the impact of MDA and/or vector control. Overall, national prevalence appears less than usually stated in the literature, especially in surveys in more recent years. Prioritization of districts by level of prevalence will strengthen the control programme, eliminate MDA in unnecessary areas and increase the likelihood of reaching the target of interruption of transmission by 2020.

## Abbreviations

GPELF: Global Programme to Eliminate Lymphatic Filariasis; ICT: Immunochromatographic test; IRS: Indoor residual spraying; ITN: Insecticide treated net; LF: Lymphatic filariasis; LLG: Local level government; LLIN: Long-lasting insecticidal net; MDA: Mass drug administration; Mf: Microfilaria (e); NRI: National Research Institute of Papua New Guinea; PacELF: Pacific Programme to Eliminate Lymphatic Filariasis; PNG: Papua New Guinea; WHO: World Health Organization.

## Competing interests

The authors declare that they have no competing interests.

## Authors’ contributions

PG: conducted literature searches, compiled data, identified survey locations, wrote manuscript; LM: managed LF programme in Papua New Guinea, conducted surveys; MS: conducted surveys, compiled data; MAB: compiled and organized data; WM: conducted surveys and lab assays; CC: PacELF Team Leader 2006–2009, managed data; ZZ & LD: organized and managed data; MO: compiled, organized and managed data; DR: conducted surveys and lab assays; KI: PacELF Team Leader 2000–2005, managed surveys and logistics; WK: checked surveys and commented on manuscript; MJB: supplied data and locations; LKH: identified locations and mapped data, wrote manuscript. All authors read and approved the final version of the manuscript.

## Supplementary Material

Additional file 1 **Table S1.** Proportion of persons tested who were positive for LF (microfilariae (Mf) or antigenemia by ICT or Og4C3 test) by survey site (all surveys conducted 1980-2011, listed in chronological order by district). Click here for file
