# Supplementary material for: Lymphatic filariasis in Papua New Guinea: distribution at district level and impact of mass drug administration, 1980 to 2011
Source: Parasit Vectors. 2013 Jan 11;6:7. doi: 10.1186/1756-3305-6-7 (PMC3606332; doi:10.1186/1756-3305-6-7)
Supplement: Additional file 1 — Table S1. Proportion of persons tested who were positive for LF (microfilariae (Mf) or antigenemia by ICT or Og4C3 test) by survey site (all surveys conducted 1980-2011, listed in chronological order by district). [file 1756-3305-6-7-S1.docx]

Graves et al. Additional Table 1

Proportion of persons tested who were positive for LF (microfilariae (Mf) or antigenemia by ICT or Og4C3 test) by survey site (all surveys conducted 1980-2011, listed in chronological order by district).

| **Province** | **District** | **Locality** | **MDA ^1^** | **Year** | **Prop pos Mf** | **N tested Mf** | **Prop pos**  **ICT** | **N tested ICT** | **Prop pos Og4C3** | **N tested Og4C3** | **Reference** |
| --- | --- | --- | --- | --- | --- | --- | --- | --- | --- | --- | --- |
| Autonomous Region of Bougainville | Central |  | 0 | 2003 |  |  | 0.11 | 272 |  |  | [[1](#_ENREF_1)] |
|  | Central | Rorovana 1 | 0 | 2006 |  |  | 0 | 330 | 0 | 415 | [[2](#_ENREF_2)] |
|  | Central | Rorovana 2 | 0 | 2006 |  |  | 0 | 92 |  |  | [[2](#_ENREF_2)] |
|  | North |  | 0 | 2003 |  |  | 0.13 | 218 |  |  | [[1](#_ENREF_1)] |
|  | North | Keakara | 0 | 2006 | 0.09 | 137 |  |  | 0.59 | 138 | [[2](#_ENREF_2)] |
|  | North | Sipai | 0 | 2006 | 0.14 | 44 |  |  | 0.29 | 45 | [[2](#_ENREF_2)] |
|  | North | Sisiapai | 0 | 2006 | 0.33 | 141 |  |  | 0.81 | 148 | [[2](#_ENREF_2)] |
|  | South |  | 0 | 2003 |  |  | 0.05 | 280 |  |  | [[1](#_ENREF_1)] |
| Central | Abau | Kupiano High School | 0 | 2000 |  |  | 0 | 250 |  |  | [[1](#_ENREF_1)] |
|  | Goilala | Tapini High School | 0 | 2000 |  |  | 0 | 250 |  |  | [[1](#_ENREF_1)] |
|  | Kairuku-Hiri | Sogeri: Yarowari High School | 0 | 2000 |  |  | 0 | 250 |  |  | [[1](#_ENREF_1)] |
|  | Kairuku-Hiri | Bereina High School | 0 | 2000 |  |  | 0 | 250 |  |  | [[1](#_ENREF_1)] |
|  | Kairuku-Hiri | Eboa | 0 | 2011 |  |  | 0 | 420 |  |  | [[3](#_ENREF_3)] |
|  | Kairuku-Hiri | Inawabui | 0 | 2011 |  |  | 0 | 142 |  |  | [[3](#_ENREF_3)] |
|  | Rigo | Kwikila High School | 0 | 2000 |  |  | 0 | 250 |  |  | [[1](#_ENREF_1)] |
|  | Rigo | Mamalo | 0 | 2011 |  |  | 0 | 161 |  |  | [[3](#_ENREF_3)] |
|  | Rigo | Hula | 0 | 2011 |  |  | 0 | 267 |  |  | [[3](#_ENREF_3)] |
|  | Rigo | Kalo | 0 | 2011 |  |  | 0 | 90 |  |  | [[3](#_ENREF_3)] |
| Chimbu (Simbu) | Chuave |  | 0 | 2004 |  |  | 0 | 250 |  |  | [[1](#_ENREF_1)] |
|  | Gumine |  | 0 | 2004 |  |  | 0.01 | 250 |  |  | [[1](#_ENREF_1)] |
|  | Karimui-Nomane |  | 0 | Not Done |  |  |  |  |  |  |  |
|  | Kerowagi |  | 0 | 2003 |  |  | 0.03 | 250 |  |  | [[1](#_ENREF_1)] |
|  | Kundiawa-Gembogl |  | 0 | 2003 |  |  | 0 | 250 |  |  | [[1](#_ENREF_1)] |
|  | Sina Sina-Yonggomugl |  | 0 | 2003 |  |  | 0 | 250 |  |  | [[1](#_ENREF_1)] |
| Eastern Highlands | Daulo |  | 0 | 2004 |  |  | 0 | 250 |  |  | [[1](#_ENREF_1)] |
|  | Goroka |  | 0 | 2003 |  |  | 0 | 250 |  |  | [[1](#_ENREF_1)] |
|  | Henganofi |  | 0 | 2003 |  |  | 0 | 250 |  |  | [[1](#_ENREF_1)] |
|  | Kainantu |  | 0 | 2003 |  |  | 0 | 250 |  |  | [[1](#_ENREF_1)] |
|  | Lufa |  | 0 | 2003 |  |  | 0 | 250 |  |  | [[1](#_ENREF_1)] |
|  | Obura-Wonenara |  | 0 | 2003 |  |  | 0 | 250 |  |  | [[1](#_ENREF_1)] |
|  | Obura-Wonenara | sentinel site | 0 | 2011 |  |  | 0 | 500 |  |  | [[4](#_ENREF_4)] |
|  | Okapa |  | 0 | 2003 |  |  | 0 | 250 |  |  | [[1](#_ENREF_1)] |
|  | Unggai-Benna |  | 0 | 2003 |  |  | 0 | 250 |  |  | [[1](#_ENREF_1)] |
|  | Unggai-Benna | sentinel site | 0 | 2011 |  |  | 0 | 500 |  |  | [[4](#_ENREF_4)] |
| East New Britain | Gazelle | Malabunga High School | 0 | 2002 |  |  | 0.1 | 250 |  |  | [[5](#_ENREF_5)] |
|  | Gazelle | George Brown High School | 0 | 2002 |  |  | 0.16 | 250 |  |  | [[5](#_ENREF_5)] |
|  | Kokopo | Vunapope High School | 0 | 2002 |  |  | 0.07 | 250 |  |  | [[1](#_ENREF_1)] |
|  | Kokopo | Kerawera Is | 0 | 2006 | 0.21 | 80 | 0.36 | 363 | 0.62 | 337 | [[2](#_ENREF_2)] |
|  | Kokopo | Utuan Is | 0 | 2006 | 0.26 | 35 | 0.54 | 84 | 0.66 | 82 | [[1](#_ENREF_1)] |
|  | Pomio | Gar | 0 | 2006 | 0.05 | 136 | 0.10 | 151 | 0.32 | 146 | [[1](#_ENREF_1)] |
|  | Pomio | Lat | 0 | 2006 | 0.18 | 112 | 0.38 | 185 | 0.58 | 170 | [[1](#_ENREF_1)] |
|  | Pomio | Mango Station | 0 | 2006 | 0.14 | 14 | 0.25 | 67 | 0.42 | 50 | [[1](#_ENREF_1)] |
|  | Rabaul | Malaguna High School | 0 | 2002 |  |  | 0.16 | 250 |  |  | [[5](#_ENREF_5)] |
| Enga | Kandep |  | 0 | Not Done |  |  |  |  |  |  |  |
|  | Kompiam |  | 0 | 2003 |  |  | 0.00 | 186 |  |  | [[1](#_ENREF_1)] |
|  | Lagaip-Porgera |  | 0 | 2003 |  |  | 0.00 | 113 |  |  | [[1](#_ENREF_1)] |
|  | Wabag |  | 0 | 2003 |  |  | 0.01 | 205 |  |  | [[1](#_ENREF_1)] |
|  | Wapenamanda |  | 0 | Not Done |  |  |  |  |  |  |  |
| East Sepik | Ambunti-Drekikir | Yauatong/ Albulum | 0 | 1984 | 0.68 | 79 |  |  |  |  | [[6](#_ENREF_6)] |
|  | Ambunti-Drekikir | Nanaha | 0 | 1985 | 0.67 | 137 |  |  |  |  | [[7](#_ENREF_7)] |
|  | Ambunti-Drekikir | Nanaha | 1 | 1985 | 0.39 | 126 |  |  |  |  | [[7](#_ENREF_7)] |
|  | Ambunti-Drekikir | Nanaha | 1 | 1985 | 0.42 | 125 |  |  |  |  | [[7](#_ENREF_7)] |
|  | Ambunti-Drekikir | Nanaha | 1 | 1986 | 0.38 | 119 |  |  |  |  | [[7](#_ENREF_7)] |
|  | Ambunti-Drekikir | 12 villages  (5 included in [[8](#_ENREF_8)]) | 0 | 1994 | 0.66 | 1666 |  |  |  |  | [[9](#_ENREF_9)] |
|  | Ambunti-Drekikir | Albulum 1  (village 2) | 0 | 1994 | 0.73 | 60 |  |  |  |  | [[8](#_ENREF_8), [10](#_ENREF_10)] |
|  | Ambunti-Drekikir | Nanaha  (village 7) | 0 | 1994 | 0.48 | 238 |  |  |  |  | [[8](#_ENREF_8), [10](#_ENREF_10)] |
|  | Ambunti-Drekikir | Penang  (village 1) | 0 | 1994 | 0.62 | 65 |  |  | 0.88 | 137 | [[8](#_ENREF_8), [10](#_ENREF_10), [11](#_ENREF_11)] |
|  | Ambunti-Drekikir | Yauatong  (village 5) | 0 | 1994 | 0.72 | 143 |  |  |  |  | [[8](#_ENREF_8), [10](#_ENREF_10)] |
|  | Ambunti-Drekikir | Ngahmbule (village 12) | 0 | 1994 | 0.34 | 343 |  |  | 0.70 | 97 | [[8](#_ENREF_8), [10](#_ENREF_10), [11](#_ENREF_11)] |
|  | Ambunti-Drekikir | Yauatong 2 (village 6) | 0 | 1994 | 0.88 | 60 |  |  | 0.87 | 60 | [[8](#_ENREF_8), [11](#_ENREF_11)] |
|  | Ambunti-Drekikir | Albulum 3  (village 4) | 0 | 1994 | 0.72 | 39 |  |  | 0.79 | 117 | [[8](#_ENREF_8), [11](#_ENREF_11)] |
|  | Ambunti-Drekikir | Albulum 2  (village 3) | 0 | 1994 | 0.85 | 156 |  |  |  |  | [[8](#_ENREF_8)] |
|  | Ambunti-Drekikir | Moihuak  (village 13) | 0 | 1994 | 0.60 | 242 |  |  | 0.61 | 150 | [[8](#_ENREF_8), [11](#_ENREF_11)] |
|  | Ambunti-Drekikir | Musungua  (village 8) | 0 | 1994 | 0.68 | 122 |  |  |  |  | [[8](#_ENREF_8)] |
|  | Ambunti-Drekikir | Musingwik (village 11) | 0 | 1994 | 0.33 | 218 |  |  | 0.97 | 72 | [[8](#_ENREF_8), [10](#_ENREF_10), [11](#_ENREF_11)] |
|  | Ambunti-Drekikir | Musilo  (village 10) | 0 | 1994 | 0.61 | 127 |  |  | 0.82 | 147 | [[8](#_ENREF_8), [11](#_ENREF_11)] |
|  | Ambunti-Drekikir | Moilenge  (village 9) | 0 | 1994 | 0.47 | 97 |  |  |  |  | [[8](#_ENREF_8)] |
|  | Ambunti-Drekikir | Musemblem (village 14) | 0 | 1994 | 0.32 | 309 |  |  |  |  | [[8](#_ENREF_8), [11](#_ENREF_11)] |
|  | Ambunti-Drekikir | Village 15 | 0 | 1994 |  |  |  |  | 0.81 | 178 | [[8](#_ENREF_8), [11](#_ENREF_11)] |
|  | Ambunti-Drekikir | Village 20 | 0 | 1994 |  |  |  |  | 0.94 | 80 | [[11](#_ENREF_11)] |
|  | Ambunti-Drekikir | Village 16 | 0 | 1994 |  |  |  |  | 0.65 | 104 | [[11](#_ENREF_11)] |
|  | Ambunti-Drekikir | Village 19 | 0 | 1994 |  |  |  |  | 0.65 | 172 | [[11](#_ENREF_11)] |
|  | Ambunti-Drekikir | Sum of 11 villages | 0 | 1994 | 0.67 | 1332 |  |  | 0.77 | 1322 | [[11](#_ENREF_11)] |
|  | Ambunti-Drekikir | Moderate transmission  4 villages **^2^** | 0 | 1994 | 0.47 | 797 |  |  |  |  | [[12](#_ENREF_12)] |
|  | Ambunti-Drekikir | High transmission  3 villages **^3^** | 0 | 1994 | 0.77 | 281 |  |  |  |  | [[12](#_ENREF_12)] |
|  | Ambunti-Drekikir | Moderate transmission  4 villages **^4^** | 0 | 1994 | 0.42 | 903 |  |  |  |  | [[12](#_ENREF_12)] |
|  | Ambunti-Drekikir | High transmission  3 villages **^5^** | 0 | 1994 | 0.76 | 243 |  |  |  |  | [[12](#_ENREF_12)] |
|  | Ambunti-Drekikir | All villages in  [[12](#_ENREF_12)] combined **^6^** | 0 | 1994 | 0.70 | 189 |  |  | 0.84 | 177 | [[13](#_ENREF_13)] |
|  | Ambunti-Drekikir | All villages in  [[12](#_ENREF_12)] combined **^7^** | 0 | 1994 | 0.30 | 44 |  |  | 0.48 | 44 | [[13](#_ENREF_13)] |
|  | Ambunti-Drekikir | Albulum 1  (village 2) | 1 | 1995 | 0.38 | 60 |  |  |  |  | [[8](#_ENREF_8), [10](#_ENREF_10)] |
|  | Ambunti-Drekikir | Nanaha  (village 7) | 1 | 1995 | 0.28 | 207 |  |  |  |  | [[8](#_ENREF_8), [10](#_ENREF_10)] |
|  | Ambunti-Drekikir | Penang  (village 1) | 1 | 1995 | 0.21 | 65 |  |  |  |  | [[8](#_ENREF_8), [10](#_ENREF_10)] |
|  | Ambunti-Drekikir | Yauatong 1 (village 5) | 1 | 1995 | 0.53 | 110 |  |  |  |  | [[8](#_ENREF_8), [10](#_ENREF_10)] |
|  | Ambunti-Drekikir | Ngahmbule (village 12) | 1 | 1995 | 0.26 | 300 |  |  |  |  | [[8](#_ENREF_8), [10](#_ENREF_10)] |
|  | Ambunti-Drekikir | Yauatong 2 (village 6) | 1 | 1995 | 0.48 | 60 |  |  |  |  | [[8](#_ENREF_8)] |
|  | Ambunti-Drekikir | Albulum 3  (village 4) | 1 | 1995 | 0.51 | 39 |  |  |  |  | [[8](#_ENREF_8)] |
|  | Ambunti-Drekikir | Albulum 2  (village 3) | 1 | 1995 | 0.35 | 156 |  |  |  |  | [[8](#_ENREF_8)] |
|  | Ambunti-Drekikir | Moihuak  (village 13) | 1 | 1995 | 0.37 | 242 |  |  |  |  | [[8](#_ENREF_8)] |
|  | Ambunti-Drekikir | Musungua  (village 8) | 1 | 1995 | 0.24 | 122 |  |  |  |  | [[8](#_ENREF_8)] |
|  | Ambunti-Drekikir | Musingwik (village 11) | 1 | 1995 | 0.23 | 218 |  |  |  |  | [[8](#_ENREF_8)] |
|  | Ambunti-Drekikir | Musilo  (village 10) | 1 | 1995 | 0.28 | 127 |  |  |  |  | [[8](#_ENREF_8)] |
|  | Ambunti-Drekikir | Moilenge  (village 9) | 1 | 1995 | 0.38 | 97 |  |  |  |  | [[8](#_ENREF_8)] |
|  | Ambunti-Drekikir | Musemblem (village 14) | 1 | 1995 | 0.11 | 309 |  |  |  |  | [[8](#_ENREF_8)] |
|  | Ambunti-Drekikir | Moderate transmission  4 villages **^8^** | 1 | 1995 | 0.21 | 756 |  |  |  |  | [[12](#_ENREF_12)] |
|  | Ambunti-Drekikir | High transmission  3 villages **^9^** | 1 | 1995 | 0.32 | 318 |  |  |  |  | [[12](#_ENREF_12)] |
|  | Ambunti-Drekikir | Moderate transmission  4 villages **^10^** | 1 | 1995 | 0.29 | 815 |  |  |  |  | [[12](#_ENREF_12)] |
|  | Ambunti-Drekikir | High transmission  3 villages **^11^** | 1 | 1995 | 0.51 | 192 |  |  |  |  | [[12](#_ENREF_12)] |
|  | Ambunti-Drekikir | Moderate transmission  4 villages **^12^** | 1 | 1996 | 0.06 | 790 |  |  |  |  | [[12](#_ENREF_12)] |
|  | Ambunti-Drekikir | High transmission  3 villages **^13^** | 1 | 1996 | 0.24 | 311 |  |  |  |  | [[12](#_ENREF_12)] |
|  | Ambunti-Drekikir | Moderate transmission  4 villages **^14^** | 1 | 1996 | 0.15 | 802 |  |  |  |  | [[12](#_ENREF_12)] |
|  | Ambunti-Drekikir | High transmission  3 villages **^15^** | 1 | 1996 | 0.42 | 253 |  |  |  |  | [[12](#_ENREF_12)] |
|  | Ambunti-Drekikir | Moderate transmission  4 villages **^16^** | 1 | 1997 | 0.01 | 819 |  |  |  |  | [[12](#_ENREF_12)] |
|  | Ambunti-Drekikir | High transmission  3 villages **^17^** | 1 | 1997 | 0.11 | 303 |  |  |  |  | [[12](#_ENREF_12)] |
|  | Ambunti-Drekikir | Moderate transmission  4 villages **^18^** | 1 | 1997 | 0.07 | 692 |  |  |  |  | [[12](#_ENREF_12)] |
|  | Ambunti-Drekikir | High transmission  3 villages **^19^** | 1 | 1997 | 0.22 | 257 |  |  |  |  | [[12](#_ENREF_12)] |
|  | Ambunti-Drekikir | Moderate transmission  4 villages **^20^** | 1 | 1998 | 0.01 | 750 |  |  |  |  | [[12](#_ENREF_12)] |
|  | Ambunti-Drekikir | High transmission  3 villages **^21^** | 1 | 1998 | 0.05 | 266 |  |  |  |  | [[12](#_ENREF_12)] |
|  | Ambunti-Drekikir | Moderate transmission  4 villages **^22^** | 1 | 1998 | 0.02 | 639 |  |  |  |  | [[12](#_ENREF_12)] |
|  | Ambunti-Drekikir | High transmission  3 villages **^23^** | 1 | 1998 | 0.11 | 165 |  |  |  |  | [[12](#_ENREF_12)] |
|  | Ambunti-Drekikir | All villages in  [[12](#_ENREF_12)] combined **^24^** | 1 | 1998 | 0.04 | 100 |  |  | 0.78 | 100 | [[13](#_ENREF_13)] |
|  | Ambunti-Drekikir | All villages in  [[12](#_ENREF_12)] combined **^25^** | 1 | 1998 | 0.06 | 100 |  |  |  |  | [[13](#_ENREF_13)] |
|  | Ambunti-Drekikir | All villages in  [[12](#_ENREF_12)] combined | 1 | 2003 | 0.01 | 535 |  |  | 0.17 | 531 | [[13](#_ENREF_13)] |
|  | Angoram |  | 0 | 1984 | 0.01 | 90 |  |  |  |  | [[1](#_ENREF_1)] |
|  | Maprik |  | 0 | 1984 | 0.01 | 200 |  |  |  |  | [[1](#_ENREF_1)] |
|  | Wewak | Brandi High School | 0 | 2002 |  |  | 0.01 | 357 |  |  | [[14](#_ENREF_14)] |
|  | Wewak | Yarapos High School | 0 | 2002 |  |  | 0.02 | 157 |  |  | [[14](#_ENREF_14)] |
|  | Wewak | Biem Island | 0 | 2010 |  |  | 0.48 | 572 |  |  | [[15](#_ENREF_15)] |
|  | Wosera Gawi |  | 0 | 2003 |  |  | 0.01 | 200 |  |  | [[1](#_ENREF_1)] |
|  | Yangoro Saussia | Kubalia | 0 | 2003 |  |  | 0.04 | 121 |  |  | [[1](#_ENREF_1)] |
| Western (Fly) | Middle Fly | Mogulu | 0 | 1990 | 0.51 | 293 |  |  | 0.82 | 300 | [[16](#_ENREF_16), [17](#_ENREF_17)] |
|  | Middle Fly | Waiwoi Falls | 0 | 1993 | 0.19 | 360 |  |  | 0.45 | 360 | [[18](#_ENREF_18)] |
|  | Middle Fly | Waiwoi Falls | 0 | 1993 | 0.52 | 485 |  |  | 0.76 | 485 | [[17](#_ENREF_17), [18](#_ENREF_18)] |
|  | Middle Fly |  | 0 | 1999 |  |  | 0.70 | 500 |  |  | [[1](#_ENREF_1)] |
|  | Middle Fly | Usukof | 1 | 1999 |  |  |  |  | 0.15 | 247 | [[17](#_ENREF_17), [19](#_ENREF_19)] |
|  | Middle Fly | Usukof | 1 | 1999 |  |  |  |  | 0.15 | 62 | [[19](#_ENREF_19)] |
|  | Middle Fly | Usukof | 1 | 1999 |  |  |  |  | 0.08 | 83 | [[19](#_ENREF_19)] |
|  | Middle Fly | Usukof | 1 | 1999 |  |  |  |  | 0.04 | 85 | [[19](#_ENREF_19)] |
|  | Middle Fly | Usukof | 1 | 1999 |  |  |  |  | 0.11 | 62 | [[19](#_ENREF_19)] |
|  | Middle Fly | Usukof | 1 | 1999 |  |  |  |  | 0.07 | 83 | [[19](#_ENREF_19)] |
|  | Middle Fly | Usukof | 1 | 1999 |  |  |  |  | 0.01 | 85 | [[19](#_ENREF_19)] |
|  | Middle Fly | Giakoret | 0 | 1999 |  |  |  |  | 0.78 | 67 | [[17](#_ENREF_17), [19](#_ENREF_19)] |
|  | Middle Fly | Giakoret | 0 | 1999 |  |  |  |  | 0.86 | 22 | [[19](#_ENREF_19)] |
|  | Middle Fly | Giakoret | 1 | 1999 |  |  |  |  | 0.28 | 25 | [[19](#_ENREF_19)] |
|  | Middle Fly | Nomad | 0 | 2000 |  |  | 0.68 | 174 | 0.83 | 262 | [[17](#_ENREF_17), [19](#_ENREF_19)] |
|  | Middle Fly | Nomad | 1 | 2000 |  |  |  |  | 0.80 | 146 | [[19](#_ENREF_19)] |
|  | North Fly |  | 0 | 1983 | 0.34 | 800 |  |  |  |  | [[20](#_ENREF_20)] |
|  | North Fly | Wangbin | 0 | 1986 | 0.13 | 38 |  |  |  |  | [[21](#_ENREF_21)] |
|  | North Fly | Finalbin | 0 | 1986 | 0.49 | 47 |  |  |  |  | [[21](#_ENREF_21)] |
|  | North Fly | Bultem | 0 | 1986 | 0.22 | 101 |  |  |  |  | [[21](#_ENREF_21)] |
|  | North Fly | Migalsim | 0 | 1986 | 0.27 | 52 |  |  |  |  | [[21](#_ENREF_21)] |
|  | North Fly | Ok Ma (Asikomban) | 0 | 1986 | 0.61 | 48 |  |  |  |  | [[21](#_ENREF_21)] |
|  | North Fly | Wangbin | 1 | 1988 | 0.02 | 49 |  |  |  |  | [[21](#_ENREF_21)] |
|  | North Fly | Finalbin | 1 | 1988 | 0.14 | 55 |  |  |  |  | [[21](#_ENREF_21)] |
|  | North Fly | Bultem | 1 | 1988 | 0.07 | 71 |  |  |  |  | [[21](#_ENREF_21)] |
|  | North Fly | Migalsim | 1 | 1988 | 0.12 | 63 |  |  |  |  | [[21](#_ENREF_21)] |
|  | North Fly | Ok Ma (Asikomban) | 1 | 1988 | 0.25 | 64 |  |  |  |  | [[21](#_ENREF_21)] |
|  | North Fly | Haidawogam | 0 | 1988 | 0.53 | 36 |  |  |  |  | [[21](#_ENREF_21)] |
|  | North Fly | Sissimakum | 0 | 1988 | 0.35 | 98 |  |  |  |  | [[21](#_ENREF_21)] |
|  | North Fly | Derongo (Kwari) | 0 | 1988 | 0.43 | 65 |  |  |  |  | [[21](#_ENREF_21)] |
|  | North Fly | Kumkit | 0 | 1988 | 0.83 | 6 |  |  |  |  | [[21](#_ENREF_21)] |
|  | North Fly | Atemkit | 0 | 1988 | 0.29 | 41 |  |  |  |  | [[21](#_ENREF_21)] |
|  | North Fly | Kavorabip | 0 | 1988 | 0.25 | 20 |  |  |  |  | [[21](#_ENREF_21)] |
|  | North Fly | Korkit | 0 | 1988 | 0.48 | 46 |  |  |  |  | [[21](#_ENREF_21)] |
|  | North Fly | Wangbin | 1 | 1990 | 0.11 | 95 |  |  |  |  | [[21](#_ENREF_21)] |
|  | North Fly | Finalbin | 1 | 1990 | 0.05 | 66 |  |  |  |  | [[21](#_ENREF_21)] |
|  | North Fly | Bultem | 1 | 1990 | 0.05 | 119 |  |  |  |  | [[21](#_ENREF_21)] |
|  | North Fly | Migalsim | 1 | 1990 | 0.02 | 95 |  |  |  |  | [[21](#_ENREF_21)] |
|  | North Fly | Ok Ma (Asikomban) | 1 | 1990 | 0.09 | 128 |  |  |  |  | [[21](#_ENREF_21)] |
|  | North Fly | Haidawogam | 1 | 1990 | 0.34 | 56 |  |  |  |  | [[21](#_ENREF_21)] |
|  | North Fly | Sissimakum | 1 | 1990 | 0.14 | 87 |  |  |  |  | [[21](#_ENREF_21)] |
|  | North Fly | Derongo (Kwari) | 1 | 1990 | 0.10 | 24 |  |  |  |  | [[21](#_ENREF_21)] |
|  | North Fly | Kumkit | 1 | 1990 | 0.35 | 20 |  |  |  |  | [[21](#_ENREF_21)] |
|  | North Fly | Atemkit | 1 | 1990 | 0.38 | 86 |  |  |  |  | [[21](#_ENREF_21)] |
|  | North Fly | Kavorabip | 1 | 1990 | 0.04 | 51 |  |  |  |  | [[21](#_ENREF_21)] |
|  | North Fly | Korkit | 1 | 1990 | 0.48 | 48 |  |  |  |  | [[21](#_ENREF_21)] |
|  | North Fly | Kwiroknai | 0 | 1990 | 0.44 | 85 |  |  |  |  | [[21](#_ENREF_21)] |
|  | North Fly | Rumginae, Senamrae, Dome | 0 | 1990 | 0.26 | 676 |  |  | 0.57 | 676 | [[16](#_ENREF_16), [17](#_ENREF_17)] |
|  | North Fly | Rumginae, Senamrae, Dome | 0 | 1991 | 0.32 | 1034 |  |  |  |  | [[16](#_ENREF_16)] |
|  | North Fly | Rumginae, Senamrae, Dome | 0 | 1992 | 0.13 | 208 |  |  |  |  | [[16](#_ENREF_16)] |
|  | North Fly | Rumginae, Senamrae, Dome | 1 | 1993 | 0.07 | 248 |  |  |  |  | [[16](#_ENREF_16)] |
|  | South Fly | Morehead High School | 0 | 2001 |  |  | 0.01 | 250 |  |  | [[1](#_ENREF_1)] |
|  | South Fly | Daru | 0 | 2001 |  |  | 0.00 | 250 |  |  | [[22](#_ENREF_22)] |
| Gulf | Kerema | Near Kerema | 0 | 1994 | 0.35 | 222 |  |  | 0.65 | 222 | [[17](#_ENREF_17)] |
|  | Kerema | Kerema High School | 0 | 2001 |  |  | 0.06 | 250 |  |  | [[1](#_ENREF_1)] |
|  | Kerema | Opau | 0 | 2010 | 0.12 | 466 | 0.43 | 466 |  |  | [[23](#_ENREF_23)] |
|  | Kikori | Ikakareta High School | 0 | 2001 |  |  | 0.03 | 250 |  |  | [[1](#_ENREF_1)] |
| Madang | Bogia |  | 0 | 2004 |  |  | 0.01 | 100 |  |  | [[24](#_ENREF_24)] |
|  | Madang | Buksak | 0 | 1986 | 0.32 | 96 |  |  |  |  | [[25](#_ENREF_25)] |
|  | Middle Ramu | 3 villages: Mangamanau, Yilu, Mamusi (Hagahai) | 0 | 1991 | 0.42 | 106 |  |  |  |  | [[26](#_ENREF_26)] |
|  | Middle Ramu | Mamusi,  Yuat River (Hagahai) | 1 | 1996 |  |  |  |  | 0.50 | 66 | [[27](#_ENREF_27)] |
|  | Middle Ramu | Mangamanau, Yuat River (Hagahai) | 1 | 1996 |  |  |  |  | 0.45 | 110 | [[27](#_ENREF_27)] |
|  | Middle Ramu | Yilu, Yuat River (Hagahai) | 1 | 1996 |  |  |  |  | 0.13 | 86 | [[27](#_ENREF_27)] |
|  | Middle Ramu |  | 0 | 2004 |  |  | 0.13 | 92 |  |  | [[1](#_ENREF_1)] |
|  | Rai Coast |  | 0 | 2004 |  |  | 0.10 | 68 |  |  | [[1](#_ENREF_1)] |
|  | Sumkar |  | 0 | 1996 |  |  |  |  | 0.10 | 133 | [[1](#_ENREF_1), [17](#_ENREF_17)] |
|  | Sumkar | Bagabag Is:  3 villages: Badilu, Madiu 1 & 2 | 0 | 1998 | 0.29 | 1026 |  |  | 0.53 | 1030 | [[28](#_ENREF_28)] |
|  | Sumkar | Bagabag Is | 0 | 1999 | 0.28 | 277 |  |  | 0.52 | 527 | [[29](#_ENREF_29)] |
|  | Sumkar | Bagabag Is | 1 | 2001 | 0.17 | 381 |  |  |  |  | [[29](#_ENREF_29)] |
|  | Sumkar | Bagabag Is | 1 | 2001 | 0.16 | 348 |  |  |  |  | [[29](#_ENREF_29)] |
|  | Usino Bundi | 4 villages | 0 | 2003 | 0.19 | 571 | 0.48 | 558 |  |  | [[30](#_ENREF_30)] |
|  | Usino Bundi | 4 villages | 1 | 2004 | 0.08 | 696 | 0.35 | 692 |  |  | [[30](#_ENREF_30)] |
|  | Usino Bundi | 4 villages | 1 | 2005 | 0.03 | 714 | 0.25 | 695 |  |  | [[30](#_ENREF_30)] |
|  | Usino Bundi | 4 villages | 1 | 2006 | 0.01 | 529 | 0.17 | 543 |  |  | [[30](#_ENREF_30)] |
| Manus | Manus |  | 0 | 2001 |  |  | 0.00 | 250 |  |  | [[1](#_ENREF_1)] |
| Milne Bay | Alotau | Buhutu valley (Sagarai):  7 villages ^26^ | 0 | 1994 | 0.24 | 75 |  |  |  |  | [[31](#_ENREF_31)] |
|  | Alotau | Buhutu valley:  6 villages ^26^ | 0 | 1995 |  |  |  |  | 0.55 | 434 | [[32](#_ENREF_32)] |
|  | Alotau | Dogura:  Aboma village | 0 | 1995 | 0.06 | 18 |  |  |  |  | [[33](#_ENREF_33)] |
|  | Alotau | Dogura:  Pova village | 0 | 1995 | 0.50 | 6 |  |  |  |  | [[33](#_ENREF_33)] |
|  | Alotau | Dogura: Dabudabu/ Kwabonaki village | 0 | 1995 | 0.09 | 23 |  |  |  |  | [[33](#_ENREF_33)] |
|  | Alotau | Dogura:  Wabiga village | 0 | 1995 | 0.18 | 17 |  |  |  |  | [[33](#_ENREF_33)] |
|  | Alotau | Dogura: Ineria/Agi/ Dibiuna village | 0 | 1995 | 0.07 | 28 |  |  |  |  | [[33](#_ENREF_33)] |
|  | Alotau | Dogura  (5 villages) | 0 | 1995 |  |  |  |  | 0.71 | 255 | [[32](#_ENREF_32)] |
|  | Alotau | Buhutu valley  (6 villages) ^26^ | 1 | 1996 |  |  |  |  | 0.36 | 100 | [[32](#_ENREF_32)] |
|  | Alotau | Dogura  (5 villages) | 1 | 1996 |  |  |  |  | 0.66 | 100 | [[32](#_ENREF_32)] |
|  | Alotau |  | 0 | 1996 | 0.23 | 212 | 0.53 | 212 | 0.52 | 212 | [[17](#_ENREF_17)] |
|  | Alotau | Near Alotau | 0 | 1998 | 0.23 | 100 | 0.38 | 100 |  |  | [[1](#_ENREF_1)] |
|  | Alotau | Kilakilana/ Upatau | 0 | 2005 |  |  | 0.33 | 270 |  |  | [[34](#_ENREF_34), [35](#_ENREF_35)] |
|  | Alotau | Gamadoudou/ Ulabo | 0 | 2005 |  |  | 0.20 | 200 |  |  | [[34](#_ENREF_34), [35](#_ENREF_35)] |
|  | Alotau | Gwavill | 0 | 2005 |  |  | 0.23 | 100 |  |  | [[34](#_ENREF_34), [35](#_ENREF_35)] |
|  | Alotau | Hanabala | 0 | 2005 |  |  | 0.18 | 106 |  |  | [[34](#_ENREF_34), [35](#_ENREF_35)] |
|  | Esa'ala | Wesley High School | 0 | 2001 |  |  | 0.18 | 250 |  |  | [[1](#_ENREF_1)] |
|  | Esa'ala | Kalologea | 0 | 2005 |  |  | 0.65 | 500 |  |  | [[34](#_ENREF_34), [35](#_ENREF_35)] |
|  | Esa'ala | Kalokalo | 0 | 2005 |  |  | 0.65 | 500 |  |  | [[34](#_ENREF_34), [35](#_ENREF_35)] |
|  | Kiriwina-Goodenough | Kiriwina High School | 0 | 2001 |  |  | 0.05 | 250 |  |  | [[1](#_ENREF_1)] |
|  | Kiriwina-Goodenough | Kavataria (Trobriand Is) | 0 | 2005 |  |  | 0.23 | 500 |  |  | [[34](#_ENREF_34), [35](#_ENREF_35)] |
|  | Kiriwina-Goodenough | Ulutuya  (Good Enough Is) | 0 | 2005 |  |  | 0.27 | 326 |  |  | [[34](#_ENREF_34), [35](#_ENREF_35)] |
|  | Samarai-Murua | Misima Is  (3 villages) | 0 | 1995 | 0.93 | 100 |  |  |  |  | [[36](#_ENREF_36)] |
|  | Samarai-Murua | Misima Is  (9 villages) | 0 | 1996 | 0.63 | 100 |  |  |  |  | [[36](#_ENREF_36)] |
|  | Samarai-Murua | Kimuta Is | 0 | 1996 |  |  |  |  | 0.39 | 50 | [[17](#_ENREF_17)] |
|  | Samarai-Murua | Russel Is | 0 | 1996 |  |  |  |  | 0.67 | 121 | [[17](#_ENREF_17)] |
|  | Samarai-Murua | Misima Is  (3 sentinel sites) | 1 | 1997 | 0.03 | 100 |  |  |  |  | [[36](#_ENREF_36)] |
|  | Samarai-Murua | Misima Is | 0 | 1997 |  |  | 0.56 | 144 | 0.53 | 144 | [[17](#_ENREF_17)] |
|  | Samarai-Murua | Paneati Is | 0 | 1997 |  |  | 0.00 | 97 |  |  | [[17](#_ENREF_17)] |
|  | Samarai-Murua | Panapompo | 0 | 1997 |  |  | 0.18 | 61 |  |  | [[17](#_ENREF_17)] |
|  | Samarai-Murua | Basalaki Is | 0 | 1998 |  |  |  |  | 0.74 | 100 | [[36](#_ENREF_36)] |
|  | Samarai-Murua | Misima Is  (3 villages and miners) | 0 | 2000 |  |  | 0.01 | 882 | 0.01 | 200 | [[36](#_ENREF_36)] |
|  | Samarai-Murua | Misima Is  (blood donors and elementary schoolchildren) | 0 | 2003 |  |  |  |  | 0.00 | 484 | [[36](#_ENREF_36)] |
|  | Samarai-Murua | Basalaki Is | 1 | 2003 | 0.01 | 100 |  |  | 0.35 | 100 | [[36](#_ENREF_36)] |
|  | Samarai-Murua | Kaubwaga | 0 | 2005 |  |  | 0.07 | 501 |  |  | [[34](#_ENREF_34), [35](#_ENREF_35)] |
|  | Samarai-Murua | Narian/ Gaibobo | 0 | 2005 |  |  | 0.06 | 502 |  |  | [[34](#_ENREF_34), [35](#_ENREF_35)] |
| Morobe | Bulolo |  | 0 | 2000 |  |  | 0.30 | 149 |  |  | [[1](#_ENREF_1)] |
|  | Finschafen |  | 0 | 2003 |  |  | 0.25 | 250 |  |  | [[1](#_ENREF_1)] |
|  | Huon |  | 0 | 2003 |  |  | 0.40 | 128 |  |  | [[1](#_ENREF_1)] |
|  | Kabwum |  | 0 | 2003 |  |  | 0.61 | 115 |  |  | [[1](#_ENREF_1)] |
|  | Lae |  | 0 | 2003 |  |  | 0.18 | 226 |  |  | [[1](#_ENREF_1)] |
|  | Markham |  | 0 | 2003 |  |  | 0.13 | 250 |  |  | [[1](#_ENREF_1)] |
|  | Menyamya |  | 0 | 2003 |  |  | 0.06 | 100 |  |  | [[1](#_ENREF_1)] |
|  | Nawae |  | 0 | 2003 |  |  | 0.28 | 250 |  |  | [[1](#_ENREF_1)] |
|  | Tewae-Siassi |  | 0 | 2001 | 0.03 | 150 |  |  |  |  | [[1](#_ENREF_1)] |
| National Capital District | Moresby North East | 9 Mile Settlement | 0 | 2011 |  |  | 0.01 | 155 |  |  | [[37](#_ENREF_37)] |
|  | Moresby North East | ATS Settlement | 0 | 2011 |  |  | 0.11 | 186 |  |  | [[37](#_ENREF_37)] |
|  | Moresby North West |  | 0 | Not Done |  |  |  |  |  |  |  |
|  | Moresby South | Kilakila | 0 | 2004 |  |  | 0.06 | 141 |  |  | [[38](#_ENREF_38)] |
|  | Moresby South | Badihagwa High School | 0 | 2004 |  |  | 0.02 | 192 |  |  | [[38](#_ENREF_38)] |
|  | Moresby South | Hanuabada -Laurabada | 0 | 2011 |  |  | 0.02 | 91 |  |  | [[37](#_ENREF_37)] |
|  | Moresby South | Hanuabada-Lahara | 0 | 2011 |  |  | 0.00 | 160 |  |  | [[37](#_ENREF_37)] |
|  | Moresby South | Pari | 0 | 2011 |  |  | 0.00 | 207 |  |  | [[37](#_ENREF_37)] |
|  | Moresby South | Baruni | 0 | 2011 |  |  | 0.00 | 228 |  |  | [[37](#_ENREF_37)] |
| New Ireland | Kavieng |  | 0 | 2001 |  |  | 0.06 | 250 |  |  | [[1](#_ENREF_1)] |
|  | Kavieng | Lovangai mission | 0 | 2006 | 0.16 | 104 |  |  | 0.62 | 119 | [[2](#_ENREF_2)] |
|  | Kavieng | Metvoe | 0 | 2006 | 0.19 | 123 |  |  | 0.53 | 131 | [[2](#_ENREF_2)] |
|  | Kavieng | Ungat | 0 | 2006 | 0.12 | 65 |  |  | 0.53 | 79 | [[2](#_ENREF_2)] |
|  | Kavieng | Vaikeb | 0 | 2006 | 0.13 | 88 |  |  | 0.52 | 89 | [[2](#_ENREF_2)] |
|  | Kavieng | Tioputuk (Metvoe, Ungat, Vaikeb) | 1 | 2011 |  |  | 0.00 | 6 |  |  | [[39](#_ENREF_39)] |
|  | Kavieng | Metvoe | 1 | 2011 |  |  | 0.18 | 247 |  |  | [[39](#_ENREF_39)] |
|  | Kavieng | Ungat | 1 | 2011 |  |  | 0.24 | 96 |  |  | [[39](#_ENREF_39)] |
|  | Kavieng | Vaikeb | 1 | 2011 |  |  | 0.08 | 168 |  |  | [[39](#_ENREF_39)] |
|  | Namanatai | Lihir Is | 0 | 1993 | 0.20 | 575 |  |  | 0.55 | 575 | [[17](#_ENREF_17)] |
|  | Namanatai | Lihir Is/Kunaie | 0 | 1993 | 0.00 | 36 |  |  |  |  | [[40](#_ENREF_40)] |
|  | Namanatai | Lihir Is/ Londolovit | 0 | 1993 | 0.06 | 69 |  |  |  |  | [[40](#_ENREF_40)] |
|  | Namanatai | Lihir Is/ Matakues | 0 | 1993 | 0.05 | 41 |  |  |  |  | [[40](#_ENREF_40)] |
|  | Namanatai | Lihir Is/Talis | 0 | 1993 | 0.25 | 105 |  |  |  |  | [[40](#_ENREF_40)] |
|  | Namanatai | Lihir Is/Wurtol | 0 | 1993 | 0.43 | 120 |  |  |  |  | [[40](#_ENREF_40)] |
|  | Namanatai | Lihir Is/Sianus | 0 | 1993 | 0.25 | 83 |  |  |  |  | [[40](#_ENREF_40)] |
|  | Namanatai | Lihir Is/Samo | 0 | 1993 | 0.38 | 50 |  |  |  |  | [[40](#_ENREF_40)] |
|  | Namanatai | Lihir Is/Lamboar | 0 | 1993 | 0.19 | 47 |  |  |  |  | [[40](#_ENREF_40)] |
|  | Namanatai | Lihir Is/Sali | 0 | 1993 | 0.26 | 42 |  |  |  |  | [[40](#_ENREF_40)] |
|  | Namanatai |  | 0 | 1996 | 0.15 | 330 |  |  | 0.32 | 330 | [[22](#_ENREF_22), [41](#_ENREF_41)] |
|  | Namanatai |  | 0 | 1998 | 0.21 | 140 | 0.32 | 140 |  |  | [[17](#_ENREF_17)] |
|  | Namanatai | Lihir Is/  East coast | 1 | 2003 |  |  | 0.08 | 3009 |  |  | [[42](#_ENREF_42)] |
|  | Namanatai | Lihir Is/  West coast | 1 | 2003 |  |  | 0.31 | 1969 |  |  | [[42](#_ENREF_42)] |
|  | Namanatai | Amfar | 0 | 2006 | 0.02 | 182 |  |  | 0.06 | 189 | [[2](#_ENREF_2)] |
|  | Namanatai | Lif | 0 | 2006 | 0.21 | 34 |  |  | 0.80 | 35 | [[2](#_ENREF_2)] |
|  | Namanatai | Malesak-Put | 0 | 2006 | 0.42 | 94 |  |  | 0.68 | 96 | [[2](#_ENREF_2)] |
|  | Namanatai | Tefa | 0 | 2006 | 0.24 | 81 |  |  | 0.74 | 82 | [[2](#_ENREF_2)] |
|  | Namanatai | Lihir Is/East coast | 1 | 2008 |  |  | 0.01 | 3799 |  |  | [[42](#_ENREF_42)] |
|  | Namanatai | Lihir Is/West coast | 1 | 2008 |  |  | 0.08 | 2464 |  |  | [[42](#_ENREF_42)] |
|  | Namanatai | Amfar | 1 | 2011 |  |  | 0 | 98 |  |  | [[39](#_ENREF_39)] |
|  | Namanatai | Sungkin | 1 | 2011 |  |  | 0 | 4 |  |  | [[39](#_ENREF_39)] |
|  | Namanatai | Ampotpot | 1 | 2011 |  |  | 0 | 71 |  |  | [[39](#_ENREF_39)] |
|  | Namanatai | Buel | 1 | 2011 |  |  | 0 | 6 |  |  | [[39](#_ENREF_39)] |
|  | Namanatai | Emo | 1 | 2011 |  |  | 0 | 45 |  |  | [[39](#_ENREF_39)] |
|  | Namanatai | Fonly | 1 | 2011 |  |  | 1 | 1 |  |  | [[39](#_ENREF_39)] |
|  | Namanatai | Gi | 1 | 2011 |  |  | 0 | 2 |  |  | [[39](#_ENREF_39)] |
|  | Namanatai | Ambabar | 1 | 2011 |  |  | 0 | 3 |  |  | [[39](#_ENREF_39)] |
|  | Namanatai | Kamunreu | 1 | 2011 |  |  | 0 | 1 |  |  | [[39](#_ENREF_39)] |
|  | Namanatai | Kapuang | 1 | 2011 |  |  | 0 | 1 |  |  | [[39](#_ENREF_39)] |
|  | Namanatai | Lavongai | 1 | 2011 |  |  | 0 | 1 |  |  | [[39](#_ENREF_39)] |
|  | Namanatai | Lif | 1 | 2011 |  |  | 0.18 | 17 |  |  | [[39](#_ENREF_39)] |
|  | Namanatai | Lufu | 1 | 2011 |  |  | 0 | 2 |  |  | [[39](#_ENREF_39)] |
|  | Namanatai | Malesak-Put | 1 | 2011 |  |  | 0.25 | 125 |  |  | [[39](#_ENREF_39)] |
|  | Namanatai | Nonu | 1 | 2011 |  |  | 0.06 | 18 |  |  | [[39](#_ENREF_39)] |
|  | Namanatai | Sumkin | 1 | 2011 |  |  | 0 | 3 |  |  | [[39](#_ENREF_39)] |
|  | Namanatai | Sumniul | 1 | 2011 |  |  | 1 | 1 |  |  | [[39](#_ENREF_39)] |
|  | Namanatai | Taunsip | 1 | 2011 |  |  | 0.5 | 2 |  |  | [[39](#_ENREF_39)] |
|  | Namanatai | Tefa | 1 | 2011 |  |  | 0.35 | 102 |  |  | [[39](#_ENREF_39)] |
| Northern (Oro) | Ijivitari |  | 0 | 2001 |  |  | 0.01 | 250 |  |  | [[1](#_ENREF_1)] |
|  | Ijivitari | Oro Bay | 0 | 2006 | 0.00 | 402 | 0.03 | 452 | 0.03 | 416 | [[2](#_ENREF_2)] |
|  | Sohe |  | 0 | 2001 |  |  | 0.01 | 250 |  |  | [[1](#_ENREF_1)] |
|  | Sohe | Saiho (Awala) | 0 | 2006 | 0.00 | 462 | 0.01 | 500 | 0.01 | 484 | [[2](#_ENREF_2)] |
| Southern Highlands | Ialibu-Pangia |  | 0 | 2004 |  |  | 0.00 | 250 |  |  | [[1](#_ENREF_1)] |
|  | Imbonggu |  | 0 | 2004 |  |  | 0.02 | 213 |  |  | [[1](#_ENREF_1)] |
|  | Kagua-Erave |  | 0 | Not Done |  |  |  |  |  |  |  |
|  | Komo-Margarima |  | 0 | Not Done |  |  |  |  |  |  |  |
|  | Koroba-Kopiago |  | 0 | Not Done |  |  |  |  |  |  |  |
|  | Mendi-Munihu |  | 0 | 2004 |  |  | 0.01 | 250 |  |  | [[1](#_ENREF_1)] |
|  | Nipa-Kutubu | Bosavi | 0 | 1987 | 0.26 | 161 |  |  |  |  | [[43](#_ENREF_43)] |
|  | Nipa-Kutubu | Waragu | 0 | 1987 | 0.59 | 160 |  |  |  |  | [[43](#_ENREF_43)] |
|  | Nipa-Kutubu | Bengoro | 0 | 1987 | 0.22 | 63 |  |  |  |  | [[43](#_ENREF_43)] |
|  | Nipa-Kutubu | Wambimisan | 0 | 1987 | 0.37 | 27 |  |  |  |  | [[43](#_ENREF_43)] |
|  | Nipa-Kutubu | Fogomaiyu | 0 | 1987 | 0.92 | 79 |  |  |  |  | [[43](#_ENREF_43)] |
|  | Nipa-Kutubu | Fogomaiyu | 1 | 1987 | 0.06 | 100 |  |  |  |  | [[43](#_ENREF_43)] |
|  | Nipa-Kutubu | Moro | 0 | 1994 | 0.37 | 181 |  |  | 0.52 | 181 | [[1](#_ENREF_1), [17](#_ENREF_17)] |
|  | Tari-Pori |  | 0 | Not Done |  |  |  |  |  |  |  |
| Western Highlands | Anglimp-South Wahgi |  | 0 | 2003 |  |  | 0.00 | 250 |  |  | [[1](#_ENREF_1)] |
|  | Dei |  | 0 | 2003 |  |  | 0.03 | 250 |  |  | [[1](#_ENREF_1)] |
|  | Jimi |  | 0 | 2003 |  |  | 0.00 | 234 |  |  | [[1](#_ENREF_1)] |
|  | Mount Hagen | Mt Hagen | 0 | 1998 |  |  | 0.00 | 200 |  |  | [[17](#_ENREF_17)] |
|  | Mount Hagen |  | 0 | 2003 |  |  | 0.02 | 250 |  |  | [[1](#_ENREF_1)] |
|  | Mul-Baiyer |  | 0 | 2003 |  |  | 0.01 | 250 |  |  | [[1](#_ENREF_1)] |
|  | North Wahgi |  | 0 | 2003 |  |  | 0.01 | 250 |  |  | [[1](#_ENREF_1)] |
|  | Tambul-Nebilyer |  | 0 | 2004 |  |  | 0.01 | 250 |  |  | [[1](#_ENREF_1)] |
| West New Britain | Kandrian-Gloucester |  | 0 | 2003 |  |  | 0.10 | 88 |  |  | [[1](#_ENREF_1)] |
|  | Kandrian-Gloucester | Giurisi | 0 | 2006 | 0.09 | 67 | 0.31 | 94 | 0.76 | 82 | [[2](#_ENREF_2)] |
|  | Kandrian-Gloucester | Kokopo | 0 | 2006 | 0.00 | 195 | 0.07 | 210 | 0.36 | 217 | [[2](#_ENREF_2)] |
|  | Kandrian-Gloucester | Malasongo | 0 | 2006 |  |  | 0.50 | 10 | 0.67 | 9 | [[2](#_ENREF_2)] |
|  | Kandrian-Gloucester | Siamatai | 0 | 2006 | 0.03 | 93 | 0.44 | 121 | 0.77 | 109 | [[2](#_ENREF_2)] |
|  | Talasea | Witu (Vitu) Island | 0 | 1994 | 0.10 | 69 |  |  | 0.38 | 69 | [[1](#_ENREF_1), [17](#_ENREF_17)] |
|  | Talasea | Vituhu | 0 | 2006 | 0.01 | 308 | 0.06 | 412 | 0.20 | 386 | [[2](#_ENREF_2)] |
| West Sepik (Sandaun) | Aitape-Lumi |  | 0 | Not Done |  |  |  |  |  |  |  |
|  | Nuku | Baro | 0 | 2002 |  |  | 0.04 | 136 |  |  | [[44](#_ENREF_44)] |
|  | Telefomin | Dapu | 0 | 2002 |  |  | 0.11 | 193 |  |  | [[44](#_ENREF_44)] |
|  | Vanimo-Green River |  | 0 | 2002 |  |  | 0.22 | 250 |  |  | [[1](#_ENREF_1)] |

1. MDA: mass drug administration: 0: no MDA prior to survey; 1: MDA prior to survey

2. Nanaha, Musungua, Musilo, Musumblem; duplicate data from single villages reported above, excluded from Table 1

3. Albulum 1, Penang, Albulum 2; duplicate data from single villages reported above, excluded from Table 1

4. Ngahmbule, Moihuak, Musingwik, Moilenge; duplicate data from single villages reported above, excluded from Table 1

5. Yauatong 1, Yauatong 2, Albulum 3; duplicate data from single villages reported above, excluded from Table 1

6. Duplicate data, excluded from Table 1

7. Duplicate data, excluded from Table 1

8. Nanaha, Musungua, Musilo, Musumblem; duplicate data from single villages reported above, excluded from Table 1

9. Albulum 1, Penang, Albulum 2; duplicate data from single villages reported above, excluded from Table 1

10. Ngahmbule, Moihuak, Musingwik, Moilenge; duplicate data from single villages reported above, excluded from Table 1

11. Yauatong 1, Yauatong 2, Albulum 3; duplicate data from single villages reported above, excluded from Table 1

12. Nanaha, Musungua, Musilo, Musumblem

13. Albulum 1, Penang, Albulum 2

14. Ngahmbule, Moihuak, Musingwik, Moilenge

15. Yauatong 1, Yauatong 2, Albulum 3

16. Nanaha, Musungua, Musilo, Musumblem

17. Albulum 1, Penang, Albulum 2

18. Ngahmbule, Moihuak, Musingwik, Moilenge

19. Yauatong 1, Yauatong 2, Albulum 3

20. Nanaha, Musungua, Musilo, Musumblem;

21. Albulum 1, Penang, Albulum 2

22. Ngahmbule, Moihuak, Musingwik, Moilenge

23. Yauatong 1, Yauatong 2, Albulum 3

24. Duplicate data, excluded from Table 1

25. Duplicate data, excluded from Table 1

26. Villages located ~60 km from Alotau

References cited:

1. PNGDOH: **National Plan and Application to PacELF.** PNG Department of Health; 2004.

2. Reeve D: **Comparative accuracy and 'field-friendly' effectiveness of diagnostic tools for lymphatic filariasis and neurocysticercosis in Papua New Guinea and Timor Leste with consideration on the impact on parasite reduction programs.** *DrPH.* James Cook University, School of Public Health, Tropical Medicine and Rehabilitation Sciences; 2010.

3. PNGDOH: **Report on LF survey in Central Province.** PNG Department of Health; 2011.

4. PNGDOH: **Report on LF survey in Eastern Highlands Province.** PNG Department of Health; 2011.

5. PNGDOH: **PNG filariasis assessment 2003.** 2003.

6. Kazura JW, Spark R, Forsyth K, Brown G, Heywood P, Peters P, Alpers M: **Parasitologic and clinical features of bancroftian filariasis in a community in East Sepik Province, Papua New Guinea.** *The American journal of tropical medicine and hygiene* 1984, **33:**1119-1123.

7. Day KP, Grenfell B, Spark R, Kazura JW, Alpers MP: **Age specific patterns of change in the dynamics of Wuchereria bancrofti infection in Papua New Guinea.** *The American journal of tropical medicine and hygiene* 1991, **44:**518-527.

8. Bockarie MJ, Alexander ND, Hyun P, Dimber Z, Bockarie F, Ibam E, Alpers MP, Kazura JW: **Randomised community-based trial of annual single-dose diethylcarbamazine with or without ivermectin against Wuchereria bancrofti infection in human beings and mosquitoes.** *Lancet* 1998, **351:**162-168.

9. Kazura JW, Bockarie M, Alexander N, Perry R, Bockarie F, Dagoro H, Dimber Z, Hyun P, Alpers MP: **Transmission intensity and its relationship to infection and disease due to Wuchereria bancrofti in Papua New Guinea.** *The Journal of infectious diseases* 1997, **176:**242-246.

10. Bockarie MJ, Ibam E, Alexander ND, Hyun P, Dimber Z, Bockarie F, Alpers MP, Kazura JW: **Towards eliminating lymphatic filariasis in Papua New Guinea: impact of annual single-dose mass treatment on transmission of Wuchereria bancrofti in East Sepik Province.** *Papua and New Guinea medical journal* 2000, **43:**172-182.

11. Tisch DJ, Hazlett FE, Kastens W, Alpers MP, Bockarie MJ, Kazura JW: **Ecologic and biologic determinants of filarial antigenemia in bancroftian filariasis in Papua New Guinea.** *The Journal of infectious diseases* 2001, **184:**898-904.

12. Bockarie MJ, Tisch DJ, Kastens W, Alexander ND, Dimber Z, Bockarie F, Ibam E, Alpers MP, Kazura JW: **Mass treatment to eliminate filariasis in Papua New Guinea.** *The New England journal of medicine* 2002, **347:**1841-1848.

13. Tisch DJ, Bockarie MJ, Dimber Z, Kiniboro B, Tarongka N, Hazlett FE, Kastens W, Alpers MP, Kazura JW: **Mass drug administration trial to eliminate lymphatic filariasis in Papua New Guinea: changes in microfilaremia, filarial antigen, and Bm14 antibody after cessation.** *The American journal of tropical medicine and hygiene* 2008, **78:**289-293.

14. PNGDOH: **Report on LF survey in East Sepik Province.** PNG Department of Health; 2002.

15. PNGDOH: **Report on LF survey in Biem Island, East Sepik Province.** PNG Department of Health/JICA; 2010.

16. Turner PF: **Filariasis in the Western Province of Papua New Guinea.** *PhD.* James Cook University, Anton Breinl Centre for Tropical Health and Medicine; 1993.

17. Melrose W, Pisters P, Turner P, Kombati Z, Selve BP, Hii J, Speare R: **Prevalence of filarial antigenaemia in Papua New Guinea: results of surveys by the School of Public Health and Tropical Medicine, James Cook University, Townsville, Australia.** *Papua and New Guinea medical journal* 2000, **43:**161-165.

18. Melrose WD: **Tropical Public Health Studies in Papua New Guinea with emphasis on Filariasis and Other Parasitic Diseases.** *DrPH.* James Cook University, School of Public Health and Tropical Medicine; 2002.

19. Kombati Z: **Epidemiological aspects of Bancroftian filariasis in Lake Murray and Nomad areas of Western Province, Papua New Guinea.** *PhD.* James Cook University, School of Public Health and Tropical Medicine; 2001.

20. Cattani J, Taufa T, Anderson W, Lourie J: **Malaria and filariasis in the Ok Tedi Region of the Star Mountains, Papua New Guinea.** *Papua and New Guinea medical journal* 1983, **26:**122-126.

21. Schuurkamp GJ: **The epidemiology of malaria and filariasis in the Ok Tedi region of Western Province, Papua New Guinea.** *PhD.* University of Papua New Guinea, 1992.

22. PNGDOH: **PNG filariasis assessment 2001.** PNG Department of Health; 2001.

23. JCU: **Report on LF baseline survey in Opau district, Gulf Province.** James Cook University; 2010.

24. PNGDOH: **Report on LF survey in Madang Province.** PNG Department of Health; 2004.

25. Burkot TR, Garner P, Paru R, Dagoro H, Barnes A, McDougall S, Wirtz RA, Campbell G, Spark R: **Effects of untreated bed nets on the transmission of Plasmodium falciparum, P. vivax and Wuchereria bancrofti in Papua New Guinea.** *Transactions of the Royal Society of Tropical Medicine and Hygiene* 1990, **84:**773-779.

26. Desowitz RS, Jenkins C, Anian G: **Bancroftian filariasis in an isolated hunter-gatherer shifting horticulturist group in Papua New Guinea.** *Bulletin of the World Health Organization* 1993, **71:**55-58.

27. Bockarie MJ, Jenkins C, Blakie WM, Lagog M, Alpers MP: **Control of lymphatic filariasis in a hunter-gatherer group in Madang Province.** *Papua and New Guinea medical journal* 2000, **43:**196-202.

28. Bockarie M, Tavul L, Kastens W, Michael E, Kazura JW: **Impact of untreated bednets on prevalence of Wuchereria bancrofti transmitted by Anopheles farauti in Papua New Guinea.** *Med Vet Entomol* 2002, **16**.

29. Bockarie MJ, Tavul L, Ibam I, Kastens W, Hazlett F, Tisch DJ, Alpers MP, Kazura JW: **Efficacy of single-dose diethylcarbamazine compared with diethylcarbamazine combined with albendazole against Wuchereria bancrofti infection in Papua New Guinea.** *The American journal of tropical medicine and hygiene* 2007, **76:**62-66.

30. Weil GJ, Kastens W, Susapu M, Laney SJ, Williams SA, King CL, Kazura JW, Bockarie MJ: **The impact of repeated rounds of mass drug administration with diethylcarbamazine plus albendazole on bancroftian filariasis in Papua New Guinea.** *PLoS neglected tropical diseases* 2008, **2:**e344.

31. Sapak P, Williams G: **Influence of bednets on LF in Buhutu valley.** *Pacific Health Dialog* 1997, **4**.

32. Sapak P, Williams G, Bryan J, Riley I: **Efficacy of mass single-dose diethylcarbamazine and DEC-fortified salt against bancroftian filariasis in Papua New Guinea six months after treatment.** *Papua and New Guinea medical journal* 2000, **43:**213-220.

33. Sapak P, Vallely A, Giuruna P, Maibani C: **Diurnal subperiodic Bancroftian filariasis in Dogura, PNG.** *Pacific Health Dialog* 1998, **5**.

34. MBP: **Mass Blood Surveys for Filariasis and Malaria - Milne Bay Province.** Alotau, Papua New Guinea: Milne Bay Administration Division of Health; 2005.

35. Capuano C: **Lymphatic Filariasis Elimination Program, Papua New Guinea; mission report.** Manila: WHO WPRO; 2007.

36. Sapak P, Melrose W, Durrheim D, Pawa F, Wynd S, Leggatt P, Taufa T, Bockarie M: **Evaluation of the lymphatic filariasis control program, Samurai Murua district, Papua New Guinea.** JCU; 2004.

37. PNGDOH: **Report on LF surveys in National Capital District.** PNG Department of Health; 2011.

38. PNGDOH: **Report on LF surveys in National Capital District.** PNG Department of Health; 2004.

39. PNGDOH: **Report on LF surveys in New Ireland Province.** PNG Department of Health; 2011.

40. Hii J, Bockarie MJ, Flew S, Genton B, Tali A, Dagoro H, Waulas B, Samson M, Alpers MP: **The epidemiology and control of lymphatic filariasis on Lihir Island, New Ireland Province.** *Papua and New Guinea medical journal* 2000, **43:**188-195.

41. PACELF: **PacELF Data Book 2003.** Suva, Fiji: PacELF; 2003.

42. Mitja O, Paru R, Hays R, Griffin L, Laban N, Samson M, Bassat Q: **The impact of a filariasis control program on Lihir Island, Papua New Guinea.** *PLoS neglected tropical diseases* 2011, **5:**e1286.

43. Prybylski D, Alto WA, Mengeap S, Odaibaiyue S: **Introduction of an integrated community-based bancroftian filariasis control program into the Mt Bosavi region of the Southern Highlands of Papua New Guinea.** *Papua and New Guinea medical journal* 1994, **37:**82-89.

44. PNGDOH: **Report on LF surveys in West Sepik (Sandaun) province.** PNG Department of Health; 2002.
